# Plant-based diets and incident metabolic syndrome: Results from a South Korean prospective cohort study

**DOI:** 10.1371/journal.pmed.1003371

**Published:** 2020-11-18

**Authors:** Hyunju Kim, Kyueun Lee, Casey M. Rebholz, Jihye Kim

**Affiliations:** 1 Department of Epidemiology, Johns Hopkins Bloomberg School of Public Health, Baltimore, Maryland, United States of America; 2 Welch Center for Prevention, Epidemiology, and Clinical Research, Johns Hopkins University, Baltimore, Maryland, United States of America; 3 Department of Medical Nutrition, Graduate School of East-West Medical Science, Kyung Hee University, Yongin, South Korea; Shanghai Jiao Tong University Affiliated Sixth People's Hospital, CHINA

## Abstract

**Background:**

Prior studies have shown that plant-based diets are associated with lower risk of cardiovascular risk factors and incident cardiovascular disease, but risks differed by quality of plant-based diets. No prospective studies have evaluated the associations between different types of plant-based diets and incident metabolic syndrome (MetS) and components of MetS. Furthermore, limited evidence exists in Asian populations who have habitually consumed a diet rich in plant foods for a long period of time.

**Methods and findings:**

Analyses were based on a community-based cohort of 5,646 men and women (40–69 years of age at baseline) living in Ansan and Ansung, South Korea (2001–2016) without MetS and related chronic diseases at baseline. Dietary intake was assessed using a validated food frequency questionnaire. Using the responses in the questionnaire, we calculated 4 plant-based diet indices (overall plant-based diet index [PDI], healthful plant-based diet index [hPDI], unhealthful plant-based diet index [uPDI], and pro-vegetarian diet index). Higher PDI score represented greater consumption of all types of plant foods regardless of healthiness. Higher hPDI score represented greater consumption of healthy plant foods (whole grains, fruits, vegetables, nuts, legumes, tea and coffee) and lower consumption of less-healthy plant foods (refined grains, potatoes, sugar-sweetened beverages, sweets, salty foods). Higher uPDI represented lower consumption of healthy plant foods and greater consumption of less-healthy plant foods. Similar to PDI, higher pro-vegetarian diet score represented greater consumption of plant foods but included only selected plant foods (grains, fruits, vegetables, nuts, legumes, potatoes). Higher scores in all plant-based diet indices represented lower consumption of animal foods (animal fat, dairy, eggs, fish/seafood, meat). Over a median follow-up of 8 years, 2,583 participants developed incident MetS. Individuals in the highest versus lowest quintile of uPDI had 50% higher risk of developing incident MetS, adjusting for demographic characteristics and lifestyle factors (hazard ratio [HR]: 1.50, 95% CI 1.31–1.71, *P*-trend < 0.001). When we further adjusted for body mass index (BMI), those in the highest quintile of uPDI had 24%–46% higher risk of 4 out of 5 individual components of MetS (abdominal obesity, hypertriglyceridemia, low high-density lipoprotein [HDL], and elevated blood pressure) (*P*-trend for all tests ≤ 0.001). Greater adherence to PDI was associated with lower risk of elevated fasting glucose (HR: 0.80, 95% CI 0.70–0.92, *P*-trend = 0.003). No consistent associations were observed for other plant-based diet indices and MetS. Limitations of the study may include potential measurement error in self-reported dietary intake, inability to classify a few plant foods as healthy and less-healthy, lack of data on vegetable oil intake, and possibility of residual confounding.

**Conclusions:**

In this study, we observed that greater adherence to diets consisting of a high intake of refined carbohydrates, sugars, and salty foods in the framework of plant-based diets was associated with an elevated risk of MetS. These results suggest that considering the quality of plant foods is important for prevention of MetS in a population that habitually consumes plant foods.

## Abstract 2

Jihye Kim and colleagues evaluate the association between plant based diets and incidence metabolic syndrome.

## Author summary

**Why was this study done?:**

- Plant-based diets have been associated with lower risk of cardiovascular risk factors and incident cardiovascular disease, but risks have differed by healthiness of plant-based diets.
- No data are available on how different types of plant-based diets are associated with risk of metabolic syndrome (MetS), which is strongly predictive of chronic diseases such as type 2 diabetes, cardiovascular disease, and shorter life span.
- Limited evidence is available in Asian populations, who have different dietary patterns than Western populations.

**What did the researchers do and find?:**

- We followed 5,646 South Korean middle-aged and older adults who provided data on their usual dietary intake, medical history, health behaviors, anthropometrics, blood samples for laboratory analyses, and diagnosis of health outcomes for 8 years (maximum of 14 years).
- South Korean adults who consumed diets high in less-healthy plant foods (refined grains, sugars, salty foods) and low in healthy plant foods (whole grains, fruits, vegetables, nuts, legumes, tea and coffee) and animal foods (animal fat, dairy, eggs, fish/seafood, meat, miscellaneous animal foods) had 50% higher risk of developing MetS.
- Such less-healthy plant-based diets were associated with 4 out of 5 components of MetS, including abdominal obesity, hypertriglyceridemia, low high-density lipoprotein (HDL), and elevated blood pressure.

**What do these findings mean?:**

- Plant-based diets high in less-healthy plant foods and low in healthy plant foods have an adverse association with MetS.
- In an Asian population that has habitually consumed a diet rich in plant foods for a long period of time, it may be important to consider the quality of plant foods for prevention of MetS.

## Introduction

Metabolic syndrome (MetS) is a cluster of conditions (abdominal obesity, high blood glucose, hypertriglyceridemia, low high-density lipoprotein cholesterol [HDL-C], and elevated blood pressure) that is strongly associated with development of type 2 diabetes, cardiovascular diseases, and shorter life span [1,2]. The prevalence of MetS has increased around the world, and one-third of US adults were estimated to have MetS in 2010 [3]. A recent systematic review found that the prevalence of MetS is high in the Asia-Pacific region as well, with 31.3% of South Korean adults having MetS [4].

Diet is an important modifiable risk factor of MetS. Several epidemiological studies have focused on whether diets high in plant foods and low in animal foods are associated with MetS, but findings have been mixed. Some studies found that individuals who restrict the intake of animal-based foods (meat, poultry, fish) have favorable metabolic profiles (lower body mass index [BMI], lower blood pressure, lower fasting glucose) [5,6], but others reported no association [7,8] or adverse associations [9–11]. However, many of these studies primarily used a cross-sectional study design and limited assessment of dietary intake to only animal foods [5,6]. Intake of plant foods, particularly less-healthy plant foods, such as refined carbohydrates or plant foods high in sugar (e.g., sugar-sweetened beverages) and salt have not been taken into account in these prior studies.

Plant-based diet indices address these limitations, as they assess intake of plant foods and animal foods, and consider healthiness of plant foods [12]. For instance, the “overall plant-based diet index” (PDI) assesses adherence to diets higher in plant foods and lower in animal foods [13–15]. Similar to PDI, the “pro-vegetarian diet index” emphasizes intake of selected plant foods and lower intake of animal foods [16]. The “healthful plant-based diet index” (hPDI) measures alignment to diets higher in healthy plant foods (fruits, vegetables, whole grains, nuts, legumes, tea and coffee) and lower in less-healthy plant foods (e.g., refined grains, foods high in sugars) and animal foods [13–15]. The “unhealthful plant-based diet index” (uPDI) is the opposite of hPDI in that it emphasizes higher intake of plants foods high in refined carbohydrates and sugars in the context of overall plant-based diets [13–15]. Prospective studies using these plant-based diet indices have found that greater adherence to PDI, hPDI, and the pro-vegetarian diet index have been associated with less weight gain and lower risk of hypertension, type 2 diabetes, chronic kidney disease, and cardiovascular disease [12–15,17]. On the contrary, greater adherence to uPDI has been associated with greater weight gain and higher risk of hypertension, type 2 diabetes, chronic kidney disease, and cardiovascular disease [12–15,17].

However, no prospective studies have used these established plant-based diet indices to assess the associations between plant-based diets and MetS. Furthermore, limited evidence on plant-based diets and chronic diseases are available in Asian populations. Given that dietary patterns of Asian populations contain higher amounts of plant foods than Western populations—and there may be differences in metabolic and genetic responses [18–20]—it is important to investigate whether associations observed in Western populations are consistent with responses in Asian populations. To our knowledge, only one prospective study on plant-based diets has been conducted in an Asian population. In Singapore, a prospective cohort study found that higher adherence to PDI and hPDI was associated with a lower risk of type 2 diabetes [21].

In the present study, we aimed to prospectively evaluate the associations between different plant-based diet indices (PDI, hPDI, uPDI, and pro-vegetarian diet) and risk of MetS in a community-based cohort of South Korean adults.

## Methods

### Study design and study population

The Korean Genome and Epidemiology Study (KoGES) is a prospective cohort study of 10,030 participants (40–69 years of age) living in Ansan and Ansung, near Seoul, South Korea [22]. Participants were recruited into the study between 2001 and 2002 (baseline) and returned for biennial follow-up visits until 2016. In KoGES, 62.2% of participants returned for the last follow-up visit conducted in 2016. The Institutional Review Boards of the Korea Centers for Disease Control and Prevention and Kyung Hee University (KHGIRB-19-398) approved the study protocol, and participants provided written informed consent. This study is reported as per the Strengthening the Reporting of Observational Studies in Epidemiology (STROBE) guideline (S1 Checklist). Our prospective analysis plan is included in S1 Text.

Of 10,030 participants, we excluded 376 participants with implausibly low or high total energy intake (<500 kcal or >5,000 kcal) and 489 participants with cardiovascular disease (myocardial infarction, stroke, or angina) or cancer because diagnosis of chronic diseases may prompt individuals to change their dietary behaviors. We then excluded 3,312 participants who had MetS at baseline. Lastly, we excluded 207 participants with missing covariates. Our final analytic sample was 5,646.

### Plant-based diet scores

At baseline and at visit 3 (2005–2006), participants’ usual intake of foods and beverages was assessed with a validated 106-item semiquantitative food frequency questionnaire [23] (S2 Text). Participants reported the frequency and the portion size of food consumption in the previous year. The food frequency questionnaire had 9 options to select from for frequency of consumption, ranging from “almost never” to “3 times per day,” and had 3 options for serving size (0.5 serving, 1 serving, and 2 servings) [24]. Nutrient intake was calculated by multiplying the amount of consumption and nutrient composition of each food using a food composition table from the Korean Nutrition Society [25].

We used cumulative average to incorporate 2 dietary assessments (averaging dietary intakes at baseline and visit 3). When participants developed MetS before visit 3 or did not complete the questionnaire at visit 3, only dietary intakes from the baseline were used.

We calculated 4 plant-based diet indices (PDI, hPDI, uPDI, and pro-vegetarian diet index) based on the responses from the food frequency questionnaire. All food items listed in the food frequency questionnaire were included in the calculation of the plant-based diet indices. A detailed description of the calculation of each diet index and differences and similarities between the indices has been described in previous papers [12,13,16]. Briefly, all food items were categorized into 17 food groups for the PDI, hPDI, and uPDI and 11 food groups for the pro-vegetarian diet index (S1 Table). These food groups were classified as healthy plant foods (whole grains, fruits, vegetables, nuts, legumes, tea and coffee), less-healthy plant foods (refined grains, potatoes, sugar-sweetened beverages, sweets and desserts, salty foods), and animal foods (animal fat, dairy, eggs, fish or seafood, meat, miscellaneous animal foods) for the PDI, hPDI, and uPDI consistent with prior studies, and plant foods (grains, fruits, vegetables, nuts, legumes, potatoes) and animal foods (animal fat, dairy, eggs, fish or seafood, meat) for the pro-vegetarian diet index. We modified the PDI, hPDI, and uPDI by adding a “salty foods” category, which included pickled and salted vegetables—which are often preserved in soy sauce or salt for a long time—to take into account high consumption of these items in this population and by removing “vegetable oil” and “fruit juices” categories because oil intake was not assessed in the questionnaire and fruit juices were asked together with fruits.

Then, for each food group, we adjusted for total energy intake using the residual method and ranked participants by quintiles [26,27]. Briefly, participants’ intakes of each food group were regressed on their total energy intake, and then residuals were calculated by subtracting predicted intakes from observed intakes. We used residuals of each food group to rank participants by quintiles, because residuals are independent of amount of food consumed. For the PDI and pro-vegetarian diet index, all plant foods were positively scored. For example, individuals in the highest quintile of vegetable consumption received a score of 5, and those in the lowest quintile received a score of 1. For the hPDI, only healthy plant foods were positively scored. For the uPDI, only less-healthy plant foods were positively scored. In all plant-based diet indices, animal foods were negatively scored. For instance, those in the highest quintile of meat consumption received a score of 1, and those in the lowest quintile received a score of 5. Thus, higher scores in all plant-based diet indices represented lower consumption of animal foods. Higher PDI score represented greater consumption of all types of plant foods regardless of healthiness. Higher hPDI score represented greater consumption of healthy plant foods and lower consumption of less-healthy plant foods. Higher uPDI score represented lower consumption of healthy plant foods and greater consumption of less-healthy plant foods. Higher pro-vegetarian diet score was similar to PDI in that it represented greater consumption of all plant foods, but different in that it did not score some food groups (tea and coffee, sugar-sweetened beverages, sweets, salty foods, miscellaneous animal foods). After adding up the scores across each food group, we divided the overall index scores into quintiles for analyses to reflect the design of the plant-based diet indices, and to be consistent with prior studies [12,28]. The theoretical range of PDI, hPDI, and uPDI was 17 to 85 and of the pro-vegetarian diet index was 11 to 55.

In the present study, PDI and pro-vegetarian diet index showed the highest Spearman correlation (*ρ* = 0.71), but other diet indices showed lower correlations with each other ranging from −0.19 between uPDI and hPDI to 0.61 between hPDI and the pro-vegetarian diet index.

### Measurement

At each visit, participants self-reported their medical history and medication use. Trained staff measured participants’ height, weight, waist circumference, blood pressure, and biochemical tests biennially. The study procedures have been described in detail previously [29]. Participants wore light clothing and had no shoes when height, weight, and waist circumference were measured to the nearest 0.1 cm or 0.1 kg. For waist circumference, the narrowest point between the lowest rib and the uppermost border of the iliac crest was measured 3 times and averaged. A Baumanometer mercury sphygmomanometer (W.A. Baum Co., Copiague, NY) was used to measure blood pressure after participants rested for 5 minutes in a sitting position using a standardized protocol. Systolic (SBP) and diastolic blood pressure (DBP) were measured in both arms twice, and values were averaged. Participants provided blood samples after ≥8 hours of fasting. At each site, blood samples were centrifuged and refrigerated at 4°C until they were transported to a central clinical laboratory. At the clinical laboratory, blood samples were stored at −80°C until analyses. An autoanalyzer (ADVIA 1650, Bayer HealthCare, Tarrytown, NY) was used to assess the concentrations of glucose, triglycerides, and HDL-C enzymatically using a standardized protocol. All instruments were calibrated before analysis using a calibrator, and only the values that were within the limit of detection were reported. A reliability study showed that laboratory assessment of these biomarkers is highly reproducible, with Pearson’s correlation >0.99 [30].

### Ascertainment of MetS

We defined MetS based on the criteria established by the National Cholesterol Education Program Adult Treatment Panel III and modified by the American Heart Association and the National Heart, Lung, and Blood Institute [2]. Incident MetS was defined as having 3 or more of the following conditions: (1) abdominal obesity (waist circumference ≥80 cm for women, or ≥90 cm for men), (2) high fasting blood glucose (fasting blood glucose ≥100 mg/dL, doctor’s diagnosis of diabetes mellitus, or diabetes medication use), (3) hypertriglyceridemia (concentration of plasma triglycerides ≥150 mg/dL), (4) low HDL-C (concentration of plasma HDL-C <50 mg/dL for women, or <40 mg/dL for men), and (5) elevated blood pressure (SBP ≥130 mmHg, DBP ≥85 mmHg, doctor’s diagnosis of hypertension, or antihypertensive medication use). Previous studies in Koreans used these criteria to define incident MetS [31,32]. Follow-up period was calculated as the time from baseline examination until the date of MetS event or censoring. We defined censoring as participants who did not return for follow-up. Participants may not have returned for follow-up due to death, but data on vital status were not available in this data set.

### Covariates

Participants completed structured questionnaires to report demographic characteristics (age, sex, education) and lifestyle factors (total energy intake, physical activity, smoking status, and alcohol intake). We categorized education level into <6 years, 7 to ≤12 years, and >12 years. We calculated metabolic equivalent of task (MET) per day for each participant by accounting for types and intensity of physical activity [33]. Smoking habits were categorized into never, former, and current smokers. Alcohol consumption (grams/day) was divided into quartiles. We calculated BMI (kilograms divided by meters squared) from measured height (centimeters) and weight (kilograms). Models were adjusted for age, total energy intake, physical activity, and BMI as continuous variables.

### Statistical analyses

All data were analyzed using STATA version 14 (StataCorp LP, College Station, TX), and *P* < 0.05 was considered statistically significant.

We examined baseline characteristics of the study population by quintiles of plant-based diet indices using ANOVA for continuous variables and chi-squared test for categorical variables. Then, we compared nutritional characteristics by quintiles of all diet indices. Macronutrients were expressed as a percentage of total energy intake. Fiber and micronutrients were expressed per 1,000 kcal.

We used 3 nested Cox proportional hazards models to evaluate the associations between plant-based diets and incident MetS. Length of follow-up time was used as the time metric. We verified that there was no violation of the proportionality assumption by assessing Schoenfeld residuals and log(-log) plots. In model 1, we adjusted for age, sex, and total energy intake. In model 2, we additionally adjusted for education, physical activity, smoking status, and alcohol intake. In model 3, we additionally adjusted for BMI. Linear trends were tested by using the median score within each quintile. We analyzed all plant-based diets continuously using per standard deviation (SD) higher to compare the indices in a standardized manner. Next, we examined whether plant-based diets were associated with individual components of MetS (abdominal obesity, high fasting glucose, hypertriglyceridemia, low HDL-C, and elevated blood pressure) in the fully adjusted models. Lastly, we tested for effect modification by sex, given that prior studies of the relationship between diet and disease reported that associations may differ between men and women [31]. However, we did not stratify the results by sex because no significant interaction was observed (*P* for interaction ≥ 0.05).

As a post hoc analysis, we visually depicted the associations between plant-based diet indices and MetS using restricted cubic splines with 4 knots at 5th, 35th, 65th, and 95th percentiles. As sensitivity analyses, we excluded individuals who developed incident cardiovascular diseases (myocardial infarction, stroke, or angina, *n* = 109) or incident diabetes (self-reported diabetes, diabetes medication use, fasting glucose ≥126 mg/dL, *n* = 90) before developing MetS.

## Results

PDI ranged from 31 to 72, hPDI ranged from 30 to 74, uPDI ranged from 30 to 74, and pro-vegetarian diet index ranged from 13 to 51 (Table 1). Those in the highest quintiles of PDI, hPDI, and pro-vegetarian diet index were more likely to be women, to be never smokers, to be more physically active, and to consume lower amounts of alcohol. In contrast, those in the highest quintile of uPDI were more likely to be men and to consume higher amounts of alcohol and less likely to be never smokers. Trends in other demographic characteristics (i.e., education) and SBP were similar for all diet indices.

**Table 1:** Baseline characteristics of KoGES according to quintiles of plant-based diet indices (*N* = 5,646)^a^.

		Quintile 1	Quintile 2	Quintile 3	Quintile 4	Quintile 5
**PDI**						
**Sample size, *n***		1,390	1,225	863	1,076	1,092
**Median score (range)**		45 (31–47)	49 (48–50)	51 (51–52)	54 (53–55)	58 (56–72)
**Women, %**		42.7	48.2	47.3	52.4	50.7
**Age (years)**		49.0 (8.0)	50.4 (8.5)	51.6 (8.9)	51.7 (8.7)	52.4 (8.9)
**Education**						
	≤6 years, %	18.8	25.2	29.5	29.6	35.8
	7–12 years, %	21.9	23.6	21.8	24.6	25.2
	>12 years, %	59.3	51.2	48.7	45.7	39.0
**Smoking status**						
	Never smoker,%	51.9	56.4	57.2	59.8	58.2
	Former smoker, %	17.0	18.1	15.1	14.8	16.6
	Current smoker, %	31.2	25.5	27.7	25.5	25.3
**BMI (kg/m**^**2**^**)**		23.8 (2.9)	23.7 (2.8)	23.7 (2.8)	23.7 (2.9)	24.0 (3.0)
**Alcohol (g/wk)**		12.5 (24.5)	10.4 (22.3)	9.8 (19.7)	8.2 (18.1)	8.6 (23.1)
**Total energy intake (kcal/d)**		2,020.2 (549.9)	1,872.4 (521.1)	1,802.7 (480.5)	1,831.1 (534.9)	1,922.8 (513.9)
**Exercise (MET/d)**		21.8 (14.4)	22.7 (14.5)	23.7 (15.2)	23.5 (14.7)	25.7 (15.8)
**Waist circumference (cm)**		80.2 (7.8)	79.8 (7.9)	79.9 (7.8)	79.3 (8.0)	80.8 (8.3)
**Fasting plasma glucose (mg/dL)**		89.3 (16.8)	89.1 (17.2)	88.5 (14.6)	87.7 (13.5)	88.5 (15.6)
**Triglycerides (mg/dL)**		124.4 (81.0)	125.1 (90.1)	128.6 (93.2)	119.8 (67.6)	123.3 (77.2)
**HDL-C (mg/dL)**		51.9 (11.8)	52.1 (11.6)	52.1 (11.6)	53.0 (12.0)	51.8 (11.5)
**SBP (mmHg)**		115.3 (16.4)	116.0 (16.8)	116.9 (17.7)	116.3 (18.1)	117.7 (17.4)
**DBP (mmHg)**		77.3 (11.3)	77.1 (11.5)	77.2 (11.2)	77.0 (11.4)	78.1 (11.3)
**Healthy plant foods (servings/d)**^**b**^		8.6 (4.1)	9.3 (4.0)	9.4 (4.0)	10.5 (4.5)	12.2 (4.8)
**Less-healthy plant foods (servings/d)**^**c**^		6.8 (2.8)	7.0 (3.0)	7.3 (2.8)	7.8 (3.1)	8.9 (3.2)
**Animal foods (servings/d)**^**d**^		4.2 (2.2)	3.5 (1.9)	3.1 (1.7)	3.1 (1.8)	2.8 (1.8)
**hPDI**						
**Sample size, *n***		1,348	1,006	1,342	866	1,084
**Median score (range)**		44 (30–46)	48 (47–49)	51 (50–53)	55 (54–56)	59 (57–74)
**Women, %**		35.2	42.9	49.1	56.7	60.2
**Age (years)**		49.4 (8.4)	50.7 (8.8)	51.1 (8.7)	51.2 (8.3)	52.3 (8.8)
**Education**						
	≤6 years, %	20.7	26.5	29.1	29.4	31.8
	7–12 years, %	22.3	23.4	22.7	25.2	24.2
	>12 years, %	57.0	50.1	48.2	45.4	44.0
**Smoking status**						
	Never smoker,%	44.0	51.1	59.2	63.5	67.5
	Former smoker, %	17.8	19.3	15.0	15.1	14.9
	Current smoker, %	38.2	29.6	25.8	21.4	17.5
**BMI (kg/m**^**2**^**)**		23.8 (2.8)	23.6 (2.8)	23.6 (2.8)	24.0 (3.0)	23.9 (3.0)
**Alcohol (g/wk)**		13.7 (25.3)	9.9 (20.7)	9.4 (20.5)	8.6 (20.6)	7.6 (21.1)
**Total energy intake (kcal/d)**		1,915.5 (543.4)	1,798.1 (537.5)	1,835.7 (505.7)	1,925.5 (504.1)	2,034.6 (521.6)
**Exercise (MET/d)**		21.9 (14.4)	23.5 (15.3)	23.9 (15.2)	23.7 (14.8)	24.2 (15.0)
**Waist circumference (cm)**		80.5 (7.8)	79.7 (7.8)	79.5 (7.6)	80.2 (8.5)	80.4 (8.3)
**Fasting plasma glucose (mg/dL)**		88.1 (11.8)	88.7 (15.8)	88.6 (15.6)	88.6 (17.6)	89.4 (18.5)
**Triglycerides (mg/dL)**		132.3 (95.6)	125.1 (89.3)	118.8 (70.7)	120.0 (72.9)	122.8 (75.8)
**HDL-C (mg/dL)**		50.8 (11.6)	52.2 (11.9)	52.4 (11.5)	53.3 (11.8)	52.6 (11.8)
**SBP (mmHg)**		115.0 (16.3)	116.2 (17.8)	117.1 (17.2)	115.6 (16.5)	117.8 (18.3)
**DBP (mmHg)**		77.1 (11.2)	77.4 (11.6)	77.5 (11.2)	77.1 (11.2)	77.7 (11.8)
**Healthy plant foods (servings/d)**^**b**^		8.1 (3.6)	8.7 (4.1)	9.7 (4.1)	11.1 (4.4)	12.7 (4.7)
**Less-healthy plant foods (servings/d)**^**c**^		9.2 (2.9)	7.8 (2.9)	7.2 (2.9)	6.7 (2.9)	6.2 (2.9)
**Animal foods (servings/d)**^**d**^		4.3 (2.0)	3.4 (2.1)	3.2 (1.8)	3.1 (1.8)	2.7 (1.6)
**uPDI**						
**Sample size, *n***		1,292	1,100	1,245	990	1,019
**Median score (range)**		43 (30–45)	48 (46–49)	52 (50–53)	55 (54–57)	60 (58–74)
**Women, %**		65.8	51.2	45.5	37.3	35.5
**Age (years)**		48.6 (7.6)	50.0 (8.4)	51.2 (8.6)	52.5 (9.1)	52.6 (9.0)
**Education**						
	≤6 years, %	13.1	23.5	28.6	36.1	38.9
	7–12 years, %	21.4	23	24.7	21.9	26.1
	>12 years, %	65.5	53.5	46.7	42.0	35.0
**Smoking status**						
	Never smoker,%	69.8	59.2	53.9	47.5	48.1
	Former smoker, %	12.5	17.5	17.3	17.2	18.4
	Current smoker, %	17.7	23.3	28.8	35.4	33.5
**BMI (kg/m**^**2**^**)**		23.9 (2.7)	23.8 (2.9)	23.7 (2.8)	23.6 (2.8)	23.9 (3.1)
**Alcohol (g/wk)**		7.6 (19.4)	9.6 (20.6)	11.0 (23.7)	11.9 (24.1)	10.7 (22.0)
**Total energy intake (kcal/d)**		1,821.2 (493.9)	1,860.9 (512.0)	1,852.1 (475.0)	1,916.0 (539.5)	2,085.2 (596.8)
**Exercise (MET/d)**		20.1 (17.8)	22.0 (13.6)	23.9 (15.4)	24.5 (15.7)	27.3 (17.2)
**Waist circumference (cm)**		78.3 (7.7)	79.5 (7.9)	80.2 (8.0)	80.7 (7.7)	81.9 (8.1)
**Fasting plasma glucose (mg/dL)**		89.3 (18.7)	88.9 (16.6)	88.1 (12.9)	89.1 (17.6)	87.8 (11.4)
**Triglycerides (mg/dL)**		113.8 (58.1)	121.6 (87.9)	125.4 (80.9)	129.9 (90.7)	132.7 (91.8)
**HDL-C (mg/dL)**		53.1 (12.0)	52.3 (11.4)	52.0 (11.3)	51.8 (12.2)	51.1 (11.4)
**SBP (mmHg)**		113.2 (16.3)	114.9 (17.2)	116.4 (16.9)	118.6 (17.8)	119.5 (17.4)
**DBP (mmHg)**		75.5 (11.6)	76.9 (11.1)	77.5 (10.9)	78.6 (11.9)	78.9 (11.0)
**Healthy plant foods (servings/d)**^**b**^		12.1 (4.1)	10.8 (4.5)	9.5 (4.1)	8.7 (4.4)	8.0 (4.2)
**Less-healthy plant foods (servings/d)**^**c**^		5.8 (2.3)	6.8 (2.6)	7.4 (2.7)	8.3 (2.9)	9.7 (3.3)
**Animal foods (servings/d)**^**d**^		4.2 (1.9)	3.7 (1.9)	3.3 (1.9)	3.0 (1.9)	2.6 (1.8)
**Pro-vegetarian diet index**						
**Sample size, *n***		1,303	1,250	928	1,183	982
**Median score (range)**		27 (13–29)	31 (30–32)	33 (33–34)	36 (35–37)	40 (38–51)
**Women, %**		32.5	45.8	50.4	55.4	60.2
**Age (years)**		48.3 (7.6)	50.1 (8.3)	50.9 (8.6)	52.0 (8.9)	53.9 (9.1)
**Education**						
	≤6 years, %	14.6	21.7	27.9	33.2	43.1
	7–12 years, %	20.1	24.4	25.3	24	23.9
	>12 years, %	65.3	53.9	46.8	42.8	33.0
**Smoking status**						
	Never smoker, %	41.9	53.3	58.7	64.6	67.5
	Former smoker, %	19.3	17.6	15.9	14.8	13.6
	Current smoker, %	38.8	29.1	25.3	20.6	18.8
**BMI (kg/m**^**2**^**)**		23.9 (2.7)	23.7 (2.8)	23.8 (2.8)	23.7 (2.9)	23.8 (3.2)
**Alcohol (g/wk)**		14.7 (25.9)	9.9 (20.4)	9.4 (22.4)	8.1 (19.3)	7.0 (20.0)
**Total energy intake (kcal/d)**		2,052.4 (529.1)	1,839.1 (513.1)	1,818.2 (508.4)	1,830.5 (507.3)	1,936.5 (550.0)
**Exercise (MET/d)**		21.2 (13.8)	23.0 (14.5)	23.7 (14.9)	23.8 (15.3)	25.9 (16.0)
**Waist circumference (cm)**		80.6 (7.6)	79.6 (7.8)	79.7 (7.8)	79.7 (8.0)	80.4 (8.7)
**Fasting plasma glucose (mg/dL)**		88.7 (14.1)	89.4 (17.9)	88.4 (16.1)	88.2 (14.2)	88.6 (16.4)
**Triglycerides (mg/dL)**		126.8 (88.8)	127.8 (89.7)	122.3 (70.8)	122.8 (79.7)	119.1 (74.4)
**HDL-C (mg/dL)**		51.0 (11.6)	51.7 (11.9)	52.5 (11.3)	53.2 (11.8)	52.7 (11.7)
**SBP (mmHg)**		114.3 (15.4)	115.5 (17.3)	115.8 (17.1)	117.7 (17.5)	119.1 (18.7)
**DBP (mmHg)**		77.0 (11.1)	77.1 (11.4)	77.1 (11.4)	77.6 (11.4)	78.0 (11.6)
**Healthy plant foods (servings/d)**^**b**^		9.3 (4.0)	9.2 (4.1)	9.7 (10.0)	10.0 (4.5)	11.9 (5.2)
**Less-healthy plant foods (servings/d)**^**c**^		8.1 (3.0)	7.2 (3.0)	7.2 (2.9)	7.2 (3.1)	7.7 (3.2)
**Animal foods (servings/d)**^**d**^		4.9 (2.1)	3.6 (1.7)	3.1 (1.6)	2.7 (1.6)	2.3 (1.5)

^a^The quintiles do not have equal sample size because many participants received the same scores.

^b^Healthy plant foods are aggregated consumption of whole grains, fruits, vegetables, nuts, legumes, and tea and coffee.

^c^Less-healthy plant foods are aggregated consumption of refined grains, potatoes, sugar sweetened beverages, sweets and desserts, and salty food group.

^d^Animal foods are aggregated consumption of animal fat, dairy, eggs, fish, meat, and miscellaneous animal foods.

**Abbreviations:** BMI, body mass index; DBP, diastolic blood pressure; HDL-C, high-density lipoprotein cholesterol; hPDI, healthful plant-based diet index; KoGES, Korean Genome and Epidemiology Study; MET, metabolic equivalent of task; PDI, overall plant-based diet index; SBP, systolic blood pressure; uPDI, unhealthful plant-based diet index

Those in the highest quintiles of all plant-based diet indices consumed higher amounts of carbohydrate as a percentage of total energy intake, lower protein, fat, and cholesterol (S2 Table). Those in the highest quintiles of PDI, hPDI, and pro-vegetarian diet index generally consumed more nutrients than those in the lowest quintiles, with higher consumption of fiber, iron, potassium, vitamin C, folate, and beta-carotene. On the contrary, those in the highest quintile of uPDI had higher total energy intake and sodium but lower amounts of fiber and micronutrients, such as calcium, phosphorous, iron, potassium, niacin, vitamin C, vitamin B-6, beta-carotene, and vitamin E. Differences in fiber intake appeared to be slightly greater across quintiles of PDI compared to other plant-based diet indices.

**Fig 1:**
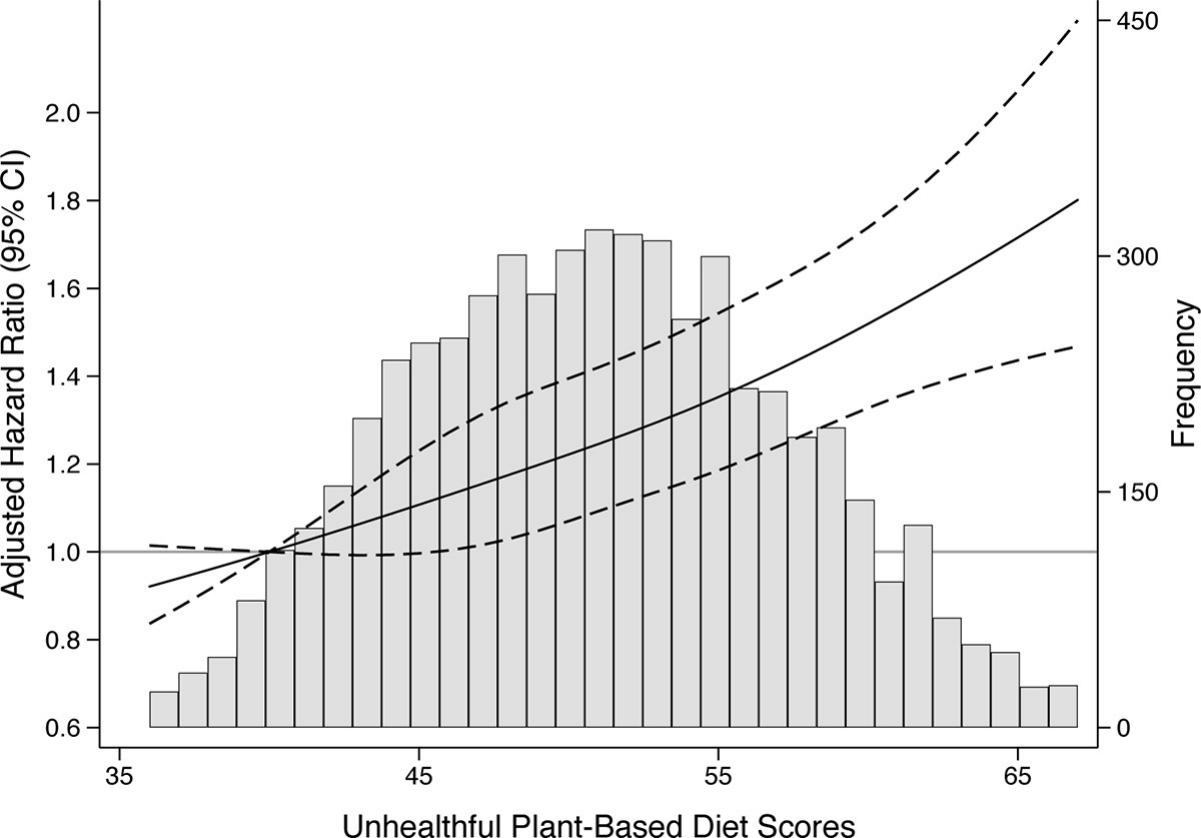
Adjusted HRs and 95% confidence intervals for incident MetS according to the continuous uPDI. The histogram in gray shows the distribution of uPDI. The solid lines represent the adjusted HRs for incident MetS, modeled using restricted cubic splines with 4 knots (5th, 35th, 65th, 95th percentiles). The reference point was set at the 5th percentile. The dashed lines represent 95% confidence intervals. HRs were adjusted for age, sex, total energy intake, education, physical activity, smoking status, alcohol intake, and BMI. BMI, body mass index; HR, hazard ratio; MetS, metabolic syndrome; uPDI, unhealthful plant-based diet index.

Over a median follow-up of 8 years, 2,583 (45.7%) participants developed incident MetS. There was a strong linear association between higher uPDI score and incident MetS (Fig 1). In model 2, those in the highest quintile of uPDI had 50% higher (hazard ratio [HR]: 1.50, 95% CI 1.31–1.71, *P*-trend < 0.001) risk of developing incident MetS compared to those in the lowest quintile of uPDI when we adjusted for demographic characteristics and lifestyle factors (Table 2). When uPDI was modeled continuously, per-1-SD higher score was associated with 16% higher (HR: 1.16, 95% CI 1.11–1.21) risk of incident MetS. This association were slightly attenuated but still remained significant when BMI was additionally adjusted. For PDI and hPDI, only individuals in quintile 4 had a lower risk of MetS relative to those in quintile 1, and this association was reflected when we visually depicted the relation between these indices with incident MetS (S1 Fig). No consistent association was observed for pro-vegetarian diet index.

**Table 2:** Prospective associations between plant-based diet indices and incident MetS (*N* = 5,646)^a^.

	Quintile 1	Quintile 2	Quintile 3	Quintile 4	Quintile 5	*P*-trend	Per SD^b^
**PDI**							
**Number of cases**	625	576	389	454	539		
**Person-years**	11,941.4	10,416.7	7,606.1	9,463.0	8,971.6		
**Model 1**	Ref	1.02 (0.91–1.14)	0.92 (0.81–1.05)	0.84 (0.75–0.95)	1.02 (0.90–1.14)	0.42	0.98 (0.94–1.02)
**Model 2**	Ref	1.02 (0.91–1.14)	0.92 (0.81–1.05)	0.85 (0.75–0.96)	1.01 (0.90–1.14)	0.37	0.98 (0.94–1.02)
**Model 3**	Ref	1.00 (0.89–1.12)	0.87 (0.76–1.00)	0.83 (0.73–0.94)	0.96 (0.82–1.04)	0.08	0.96 (0.92–1.00)
**hPDI**							
**Number of cases**	590	455	619	384	535		
**Person-years**	10,972.9	8,854.5	11,843.7	7,728.5	8,972.2		
**Model 1**	Ref	0.91 (0.81–1.03)	0.90 (0.80–1.01)	0.84 (0.74–0.96)	0.95 (0.85–1.07)	0.35	0.97 (0.93–1.01)
**Model 2**	Ref	0.93 (0.82–1.05)	0.92 (0.82–1.03)	0.86 (0.75–0.98)	0.98 (0.87–1.11)	0.63	0.98 (0.94–1.02)
**Model 3**	Ref	0.94 (0.83–1.07)	0.93 (0.83–1.04)	0.82 (0.72–0.94)	0.97 (0.86–1.10)	0.37	0.96 (0.93–1.00)
**uPDI**							
**Number of cases**	525	475	577	417	535		
**Person-years**	12,176.9	10,068.0	10,612.8	8,021.9	7,492.2		
**Model 1**	Ref	1.08 (0.95–1.22)	1.23 (1.09–1.39)	1.31 (1.15–1.49)	1.57 (1.39–1.79)	<0.001	1.18 (1.14–1.23)
**Model 2**	Ref	1.05 (0.93–1.19)	1.18 (1.05–1.33)	1.23 (1.08–1.41)	1.50 (1.31–1.71)	<0.001	1.16 (1.11–1.21)
**Model 3**	Ref	1.03 (0.91–1.17)	1.18 (1.04–1.33)	1.26 (1.11–1.44)	1.44 (1.26–1.64)	<0.001	1.15 (1.11–1.21)
**Pro-vegetarian diet index**							
**Number of cases**	565	557	396	551	514		
**Person-years**	11,466.1	11,011.1	8,178.5	10,131.7	7,584.4		
**Model 1**	Ref	0.97 (0.86–1.09)	0.89 (0.78–1.02)	0.97 (0.86–1.10)	1.11 (0.98–1.26)	0.11	1.03 (0.99–1.07)
**Model 2**	Ref	0.98 (0.87–1.10)	0.90 (0.79–1.03)	0.98 (0.87–1.11)	1.11 (0.98–1.26)	0.12	1.03 (0.99–1.07)
**Model 3**	Ref	1.01 (0.89–1.13)	0.90 (0.79–1.03)	1.00 (0.89–1.13)	1.10 (0.97–1.25)	0.22	1.02 (0.98–1.06)

Model 1 was adjusted for age, sex, and total energy intake. Model 2 was adjusted for age, sex, total energy intake, education, physical activity, smoking status, and alcohol intake. Model 3 was adjusted for age, sex, total energy intake, education, physical activity, smoking status, alcohol intake, and BMI.

^a^The quintiles do not have equal sample size because many participants received the same scores.

^b^In the continuous analysis, expressed per SD higher score, PDI had an SD of 5.3, hPDI had an SD of 6.3, uPDI had an SD of 6.9, and pro-vegetarian diet index had an SD of 5.0.

**Abbreviations:** BMI, body mass index; hPDI, healthful plant-based diet index; MetS, metabolic syndrome; PDI, overall plant-based diet index; SD, standard deviation; uPDI, unhealthful plant-based diet index

In the fully adjusted models, greater adherence to uPDI was significantly associated with 4 out of 5 individual components of MetS (Table 3). Those in the highest quintile of uPDI had 46% higher (HR: 1.46, 95% CI 1.25–1.71, *P*-trend < 0.001) risk of developing abdominal obesity, 26% higher (HR: 1.26, 95% CI 1.08–1.46, *P*-trend = 0.001) risk of developing hypertriglyceridemia, 25% higher (HR: 1.25, 95% CI 1.09–1.43, *P*-trend = 0.001) risk of developing low HDL-C, and 24% higher (HR: 1.24, 95% CI 1.06–1.45, *P*-trend = 0.001) risk of developing elevated blood pressure relative to those in the lowest quintile of uPDI.

**Table 3:** Prospective associations between plant-based diet indices and individual components of incident MetS (*N* = 5,646).

	Quintile 1	Quintile 2	Quintile 3	Quintile 4	Quintile 5	*P*-trend
**PDI**						
**Abdominal obesity**	Ref	0.99 (0.86–1.13)	0.98 (0.84–1.14)	0.96 (0.84–1.11)	0.95 (0.82–1.09)	0.41
**Hypertriglyceridemia**	Ref	1.02 (0.89–1.16)	0.99 (0.86–1.15)	0.95 (0.83–1.09)	0.98 (0.85–1.13)	0.59
**Low HDL-C**	Ref	1.11 (0.98–1.25)	1.10 (0.97–1.25)	1.06 (0.94–1.20)	1.13 (0.99–1.27)	0.15
**High fasting glucose**	Ref	0.89 (0.78–1.01)	0.92 (0.79–1.06)	0.85 (0.74–0.97)	0.80 (0.70–0.92)	0.003
**Elevated blood pressure**	Ref	0.97 (0.84–1.11)	0.95 (0.82–1.11)	0.97 (0.84–1.12)	1.01 (0.87–1.16)	0.97
**hPDI**						
**Abdominal obesity**	Ref	1.01 (0.87–1.17)	1.04 (0.91–1.19)	0.97 (0.83–1.13)	1.04 (0.89–1.20)	0.87
**Hypertriglyceridemia**	Ref	0.95 (0.82–1.09)	0.88 (0.77–1.00)	0.84 (0.72–0.97)	1.00 (0.87–1.15)	0.65
**Low HDL-C**	Ref	0.92 (0.81–1.05)	0.97 (0.87–1.09)	0.98 (0.86–1.11)	1.08 (0.95–1.22)	0.15
**High fasting glucose**	Ref	1.00 (0.87–1.14)	0.97 (0.85–1.11)	0.89 (0.77–1.04)	1.01 (0.88–1.16)	0.79
**Elevated blood pressure**	Ref	0.98 (0.85–1.13)	1.06 (0.93–1.21)	0.98 (0.84–1.14)	0.93 (0.80–1.07)	0.31
**uPDI**						
**Abdominal obesity**	Ref	1.01 (0.87–1.16)	1.26 (1.10–1.45)	1.14 (0.98–1.34)	1.46 (1.25–1.71)	<0.001
**Hypertriglyceridemia**	Ref	0.94 (0.82–1.08)	1.05 (0.91–1.20)	1.13 (0.97–1.31)	1.26 (1.08–1.46)	0.001
**Low HDL-C**	Ref	0.96 (0.85–1.09)	1.06 (0.94–1.20)	1.20 (1.05–1.36)	1.25 (1.09–1.43)	0.001
**High fasting glucose**	Ref	1.03 (0.90–1.19)	0.93 (0.81–1.08)	0.97 (0.83–1.13)	1.06 (0.91–1.24)	0.31
**Elevated blood pressure**	Ref	0.99 (0.85–1.15)	1.04 (0.90–1.21)	1.14 (0.98–1.34)	1.24 (1.06–1.45)	0.001
**Pro-vegetarian diet index**						
**Abdominal obesity**	Ref	0.90 (0.78–1.03)	0.97 (0.83–1.13)	0.93 (0.81–1.08)	0.99 (0.84–1.15)	0.92
**Hypertriglyceridemia**	Ref	0.90 (0.79–1.03)	0.93 (0.80–1.07)	0.93 (0.81–1.07)	1.02 (0.88–1.18)	0.68
**Low HDL-C**	Ref	1.06 (0.94–1.20)	1.02 (0.90–1.17)	1.16 (1.00–1.31)	1.12 (0.98–1.28)	0.07
**High fasting glucose**	Ref	0.92 (0.80–1.05)	0.96 (0.83–1.10)	0.91 (0.79–1.05)	0.93 (0.80–1.08)	0.38
**Elevated blood pressure**	Ref	0.93 (0.81–1.07)	0.90 (0.77–1.05)	0.92 (0.80–1.06)	0.99 (0.85–1.16)	0.92

Model was adjusted for age, sex, total energy intake, education, physical activity, smoking status, alcohol intake, and BMI.

^1^The quintiles do not have equal sample size because many participants received the same scores.

**Abbreviations:** BMI, body mass index; HDL-C, high-density lipoprotein cholesterol; hPDI, healthful plant-based diet index; MetS, metabolic syndrome; PDI, overall plant-based diet index; uPDI, unhealthful plant-based diet index

When we examined components of MetS, greater adherence to PDI was associated with 20% lower (HR: 0.80, 95% CI 0.70–0.92, *P*-trend = 0.003) risk of developing high fasting glucose. No consistent associations between other plant-based diet indices and MetS were observed

### Sensitivity analyses

When we excluded individuals who developed incident cardiovascular disease or diabetes during follow-up, the associations did not change substantially in the fully adjusted model (HR for hPDI_quintile 5 vs. quintile 1_: 0.98, 95% CI 0.87–1.11, *P*-trend = 0.47; HR for uPDI_quintile 5 vs. quintile 1_: 1.48, 95% CI 1.29–1.69, *P*-trend < 0.001; HR for pro-vegetarian_quintile 5 vs. quintile 1_: 1.12, 95% CI 0.99–1.27, *P*-trend = 0.12). The trend was significant for PDI, but the HR for quintile 5 versus quintile 1 was not significant (HR for PDI_quintile 5 vs. quintile 1_: 0.93, 95% CI 0.83–1.05, *P*-trend = 0.04).

## Discussion

In a community-based cohort of South Korean adults, greater adherence to less-healthy plant-based diets (diets high in refined carbohydrates, sugars, and salted vegetables and low in healthy plant foods and animal foods, captured by uPDI) was associated with a higher risk of incident MetS, adjusting for demographic characteristics and lifestyle factors. This association remained significant when BMI was additionally adjusted, and greater adherence to plant-based diets rich in less-healthy plant foods was consistently associated with individual components of MetS (abdominal obesity, hypertriglyceridemia, low HDL-C, elevated blood pressure). Higher adherence to PDI was associated with a lower risk of high fasting glucose. There was no association between other types of plant-based diets (hPDI and pro-vegetarian diet) and MetS.

Our findings on uPDI and incident MetS are generally in agreement with prior studies conducted in Western populations. In the Nurses’ Health Study, Nurses’ Health Study 2, and Health Professionals Follow-Up Study, a 1-SD increase in uPDI was associated with greater weight gain and higher risk of incident type 2 diabetes [15]. In the Atherosclerosis Risk in Communities (ARIC) Study, a community-based cohort of generally healthy US adults, those in the highest quintile of uPDI had a higher risk of incident hypertension [12]. However, our findings differed from a study by Spanish university graduates that found that an unhealthy plant-based food pattern developed from their own cohort was not associated with incident obesity [28]. Our results add to these existing studies by showing that consumption of less-healthy plant foods in the framework of overall plant-based diets may be associated with a more proximal risk factor to these cardiovascular risk factors among populations who habitually consume a diet that is rich in plant foods for a long period.

The nutrition composition of uPDI may explain how greater adherence to uPDI may be associated with incident MetS. Those in the highest quintile of uPDI consumed higher total energy intake and sodium but lower amounts of fiber, micronutrients, and antioxidants. Fiber plays an important role in carbohydrate metabolism and weight gain, and fiber intake has been associated with lower risk of inflammation and MetS [34–36]. Although we did not have data on added sugars, previous studies have shown that those with the greatest adherence to uPDI had higher added sugar intake [12]. High intake of added sugars can lead to poor glycemic control and lipid metabolism, particularly triglycerides as excess sugars can increase hepatic de novo lipogenesis [37,38]. Lower intake of micronutrients and antioxidants (potassium, vitamin A or C) can play a role in modulating endothelial dysfunction, which can lead to oxidative stress [39]. A prospective study in the US reported that women with high nutritional risk profiles characterized as having a higher dietary intake of lipids and a lower dietary intake of micronutrients (vitamin A, C, E, folate) had an elevated risk of incident MetS [40]. Excessive intake of sodium can increase blood pressure, thereby elevating the risk of MetS [41,42]. Importantly, these individual nutrients may have had synergistic effects, and our approach utilizing a predefined index allowed us to consider the totality of the diet.

We did not find an association between hPDI, pro-vegetarian diet, and overall MetS or individual components of MetS. Prior studies in Western populations reported that greater adherence to these plant-based diet indices was associated with lower risk of various cardiovascular risk factors and cardiovascular disease [12–15,17]. However, in South Koreans, intake of plant foods is higher and intake of red and processed meat is lower than in Western populations, considering that grains and vegetables are included in every meal [18,43]. It is possible that higher plant food intake in a population that is already consuming a plant-based diet may not elicit clinically significant metabolic responses. In addition, the null associations that we observed with hPDI may be due to a different categorization of foods in the present study, because several less-healthy plant foods could not be separated from healthy plant foods (i.e., fruit juices were added to the “fruits” category).

Greater adherence to PDI was associated with a lower risk of high fasting glucose, whereas other types of plant-based diets did not show an association with this MetS component. This finding is consistent with a recent meta-analysis that found that greater adherence to plant-based diets, such as PDI, are associated with lower risk of type 2 diabetes [44]. We may not have found associations with other plant-based diet indices because PDI appeared to have the greatest difference in intake of dietary fiber across quintiles (1.3 g/1,000 kcal) compared with other plant-based diet indices. Such small differences underscore that dietary patterns captured with plant-based diet indices in our analytic study population may be slightly different from what was captured in Western populations, considering the very high carbohydrate intake and low fat intake among those in quintiles 2–5 of all plant-based diets (>70% of total energy from carbohydrate and 9%–11% of total energy from fat) in our study. Thus, further research in populations with similar dietary behaviors as the South Korean population is warranted on whether other types of plant-based diets such as hPDI and a pro-vegetarian diet are associated with lower risk of MetS and high fasting glucose specifically.

The novelty of our study comes from the prospective evaluation of the associations between different types of plant-based diets and risk of MetS. Our results expand the understanding of how an unhealthful plant-based diet may be associated with type 2 diabetes and cardiovascular disease by investigating a proximal risk factor for these conditions. Strengths of our study include the use of data from a community-based cohort, validated food frequency questionnaire, repeated dietary assessments, and sufficient follow-up period to ascertain incident MetS. Our data also contribute to the literature with a unique focus on an Asian population.

However, several limitations need to be taken into account. Although we used a food frequency questionnaire validated in South Korean adults, reporting of dietary intake can still be subject to measurement error. We made slight changes to the categorization of foods, because certain less-healthy plant foods and healthy plant foods were asked together. This may have led to an attenuation of the association between hPDI and incident MetS. Furthermore, there were no data on vegetable oil or olive oil intake in this population, which is likely an important source of dietary fatty acids and could have affected blood lipid levels. Future studies could use more detailed food frequency questionnaires by asking about healthy plant foods and less-healthy plant foods separately, and assessing vegetable oil intake. Lastly, although we adjusted for important confounders, there may still be residual confounding.

The dietary guidelines for South Koreans have recommended eating a balanced diet, including a variety of foods such as grains, vegetables, fruits, beans, fish, eggs, meat, poultry, and dairy products; consuming less salt-preserved foods; using less salt when preparing foods; and selecting foods lower in salt, sugar, and fat [45]. Our findings on uPDI provide support for these recommendations, considering the strong positive association between this dietary pattern (high in refined grains, sugars, and salty foods) and incident MetS. If our findings are replicated with different health outcomes or study designs, it may be useful to make a distinction between healthy plant foods and less-healthy plant foods in future dietary guidelines for South Koreans.

In conclusion, in a community-based cohort of South Korean adults, diets high in less-healthy plant foods in the context of a plant-based diet were associated with higher risk of incident MetS, particularly abdominal obesity, hypertriglyceridemia, low HDL-C, and elevated blood pressure. These results highlight the importance of considering the quality of plant foods with regard to more-healthy plant foods versus relatively less-healthy plant foods for the prevention of MetS. Further research confirming the associations between PDI, hPDI, pro-vegetarian diet, and MetS in other ethnic populations with different dietary behaviors is warranted.

## Supporting information

S1 Checklist STROBE checklist. Note: An Explanation and Elaboration article discusses each checklist item and gives methodological background and published examples of transparent reporting. The STROBE checklist is best used in conjunction with this article (freely available on the websites of *PLOS Medicine* (http://www.plosmedicine.org/), *Annals of Internal Medicine* (http://www.annals.org/), and *Epidemiology* (http://www.epidem.com/). Information on the STROBE Initiative is available at www.strobe-statement.org.
(DOCX)

S1 Fig Adjusted HRs and 95% confidence intervals for incident MetS according to the continuous PDI, hPDI, and pro-vegetarian diet index using restricted cubic splines with 4 knots at the 5th, 35th, 65th, and 95th percentiles. The histogram in gray shows the distribution of plant-based diet scores. The solid lines represent the adjusted HRs for incident MetS, modeled using restricted cubic splines with 4 knots (5th, 35th, 65th, 95th percentiles). The reference point was set at the 5th percentile of each score. The dashed lines represent 95% confidence intervals. HRs were adjusted for age, sex, total energy intake, education, physical activity, smoking status, alcohol intake, and BMI.
(DOCX)

S1 Table Scoring system and classification of food items in KoGES. ^1^The PDI, hPDI, and uPDI categorized food groups into “healthy plant foods,” “less healthy plant foods,” and “animal foods.” The pro-vegetarian diet index categorized food groups into “plant foods” and “animal foods.” Positive indicates that higher intakes received higher scores. Reverse indicates that higher intakes received lower scores. ^2^ Whole grains and refined grains were aggregated to a “grains” food group in the pro-vegetarian diet index. hPDI, healthful plant-based diet index; PDI, overall plant-based diet index; uPDI, unhealthful plant-based diet index
(DOCX)

S2 Table Nutritional characteristics of diet according to quintiles of plant-based diet indices. (DOCX)

S1 Text Prospective analysis plan. (PDF)

S2 Text Copy of the food frequency questionnaire. (PDF)

## Abbreviations

ARIC: Atherosclerosis Risk in Communities study
BMI: body mass index
DBP: diastolic blood pressure
HDL-C: high-density lipoprotein cholesterol
hPDI: healthful plant-based diet index
HR: hazard ratio
KoGES: Korean Genome and Epidemiology Study
MET: metabolic equivalent of task
MetS: metabolic syndrome
PDI: overall plant-based diet index
SBP: systolic blood pressure
STROBE: Strengthening the Reporting of Observational Studies in Epidemiology
uPDI: unhealthful plant-based diet index
