# Supplementary material for: Plant-based diets and incident metabolic syndrome: Results from a South Korean prospective cohort study
Source: PLoS Med. 2020 Nov 18;17(11):e1003371. doi: 10.1371/journal.pmed.1003371 (PMC7673569; doi:10.1371/journal.pmed.1003371)
Supplement: S1 Checklist — Note: An Explanation and Elaboration article discusses each checklist item and gives methodological background and published examples of transparent reporting. The STROBE checklist is best used in conjunction with this article (freely available on the websites of PLOS Medicine (http://www.plosmedicine.org/), Annals of Internal Medicine (http://www.annals.org/), and Epidemiology (http://www.epidem.com/). Information on the STROBE Initiative is available at www.strobe-statement.org. (DOCX) [file pmed.1003371.s001.docx]

STROBE Statement—checklist of items that should be included in reports of observational studies

|  | Item No. | Recommendation | Page  No. | Relevant text from manuscript |
| --- | --- | --- | --- | --- |
| **Title and abstract** | 1 | (*a*) Indicate the study’s design with a commonly used term in the title or the abstract | Title  Abstract, paragraph 2 | Plant-based diets and incident metabolic syndrome: Results from a Korean prospective cohort study  Analyses were based on a community-based cohort of 5,646 middle-aged men and women adults living in Ansan and Ansung, South Korea (2001-2016) without MetS and related chronic diseases at baseline. |
|  |  | (*b*) Provide in the abstract an informative and balanced summary of what was done and what was found | Abstract paragraph 2  Abstract paragraph 3 | Analyses were based on a community-based cohort of 5,646 middle-aged men and women adults living in Ansan and Ansung, South Korea (2001-2016) without MetS and related chronic diseases at baseline.  Individuals in the highest vs. lowest quintile of uPDI had 50% higher risk of developing incident MetS, adjusting for demographic characteristics, and lifestyle factors (hazard ratio [HR]: 1.50, 95% CI: 1.31, 1.71, P-trend<0.001). When we further adjusted for BMI, those in the highest quintile of uPDI had 24-46% higher risk of 4 out of 5 individual components of MetS (abdominal obesity, hypertriglyceridemia, low high density lipoprotein, and elevated blood pressure) (P-trend for all tests ≤0.001). Greater adherence to PDI was associated with lower risk of elevated fasting glucose (HR: 0.80, 95% CI: 0.70, 0.92, P-trend=0.003). No consistent associations were observed for other plant-based diet indices and MetS. |
| Introduction | | | |  |
| Background/rationale | 2 | Explain the scientific background and rationale for the investigation being reported | Introduction, paragraph 1,2 | Metabolic syndrome (MetS) is a cluster of conditions [abdominal obesity, high blood glucose, hypertriglyceridemia, low HDL cholesterol (HDL-C), and elevated blood pressure] that is strongly associated with development of type 2 diabetes, cardiovascular diseases, and shorter life span [1,2]. …  Diet is an important modifiable risk factor of MetS. Several epidemiological studies have focused on whether diets high in plant foods and low in animal foods are associated with MetS, but findings have been mixed. Some studies found that individuals who restrict the intake of animal-based foods (meat, poultry, fish) have favorable metabolic profiles (lower body mass index, lower blood pressure, lower fasting glucose) [5,6], but others reported no association [7,8], or adverse associations [9–11]. However, many of these studies primarily used a cross-sectional study design, and limited assessment of dietary intakes to only animal foods [5,6]. Intakes of plant foods, particularly less healthy plant foods, such as refined carbohydrates or plant foods high in sugar (e.g., sugar-sweetened beverages) and salt have not been taken into account in these prior studies. |
| Objectives | 3 | State specific objectives, including any prespecified hypotheses | Introduction, paragraph 5 | In the present study, we aimed to prospectively evaluate the associations between different plant-based diet indices (PDI, hPDI, uPDI, and provegetarian diet) and risk of MetS in a community-based cohort of South Korean adults. |
| Methods | | | |  |
| Study design | 4 | Present key elements of study design early in the paper | Methods, paragraph 1 | The Korean Genome and Epidemiology Study (KoGES) is a prospective cohort study of 10,030 participants (40-69 years of age) living in Ansan and Ansung, near Seoul, South Korea [22]. Participants were recruited into the study between 2001 and 2002 (baseline) and returned for biennial follow-up visits until 2016. In KoGES, 62.2% of participants returned for the last follow-up visit conducted in 2016. The Institutional Review Boards of the Korea Centers for Disease Control and Prevention and Kyung Hee University (KHGIRB-19-398) approved the study protocol, and participants provided written informed consent. This study is reported as per the Strengthening the Reporting of Observational Studies in Epidemiology (STROBE) guideline (S1 Checklist). Our prospective analysis plan is included in Supporting Information. |
| Setting | 5 | Describe the setting, locations, and relevant dates, including periods of recruitment, exposure, follow-up, and data collection | Methods, paragraph 1 | The Korean Genome and Epidemiology Study (KoGES) is a prospective cohort study of 10,030 participants (40-69 years of age) living in Ansan and Ansung, near Seoul, South Korea [22]. Participants were recruited into the study between 2001 and 2002 (baseline) and returned for biennial follow-up visits until 2016. |
| Participants | 6 | (*a*) *Cohort study*—Give the eligibility criteria, and the sources and methods of selection of participants. Describe methods of follow-up  *Case-control study*—Give the eligibility criteria, and the sources and methods of case ascertainment and control selection. Give the rationale for the choice of cases and controls  *Cross-sectional study*—Give the eligibility criteria, and the sources and methods of selection of participants | Methods, paragraph 1 | Participants were recruited into the study between 2001 and 2002 (baseline) and returned for biennial follow-up visits until 2016. In KoGES, 62.2% of participants returned for the last follow-up visit conducted in 2016. |
|  |  | (*b*) *Cohort study*—For matched studies, give matching criteria and number of exposed and unexposed  *Case-control study*—For matched studies, give matching criteria and the number of controls per case |  |  |
| Variables | 7 | Clearly define all outcomes, exposures, predictors, potential confounders, and effect modifiers. Give diagnostic criteria, if applicable | Methods, paragraph 3-6  Methods, paragraph 9 | At baseline and at visit 3 (2005-2006), participants’ usual intake of foods and beverages was assessed with a validated 106-item semi-quantitative food frequency questionnaire [23] (Supporting Information)….  We defined MetS based on the criteria established by the National Cholesterol Education Program Adult Treatment Panel III, and modified by the American Heart Association and the National Heart, Lung, and Blood Institute [2]. Incident MetS was defined as having 3 or more of the following conditions:… |
| Data sources/ measurement | 8* | For each variable of interest, give sources of data and details of methods of assessment (measurement). Describe comparability of assessment methods if there is more than one group | Methods, paragraph 3  Methods, paragraph 8  Methods, paragraph 10 | Plant-based diet scores: At baseline and at visit 3 (2005-2006), participants’ usual intake of foods and beverages was assessed with a validated 106-item semi-quantitative food frequency questionnaire [23] (Supporting Information)….  Measurement: Participants self-reported medical history and medication use. Trained staff measured participants’ height, weight, waist circumference, blood pressure, and biochemical tests biennially. The study procedures have been described in detail previously [28]….  Covariates: Participants completed structured questionnaires to report demographic characteristics (age, sex, education) and lifestyle factors (total energy intake, physical activity, smoking status, and alcohol intake)…. |
| Bias | 9 | Describe any efforts to address potential sources of bias | Methods, paragraph 12  Methods, paragraph 13 | In model 1, we adjusted for age, sex, and total energy intake. In model 2, we additionally adjusted for total energy intake, education, physical activity, smoking status, and alcohol intake. In model 3, we additionally adjusted for BMI. Linear trends were tested by using the median score within each quintile. …  As a post-hoc analysis, we … additionally adjusted for incident cardiovascular diseases (myocardial infarction, stroke, or angina, n=109) and incident diabetes (self-reported diabetes, diabetes medication use, fasting glucose ≥126 mg/dL, n=90) which occurred before developing MetS as covariates. |
| Study size | 10 | Explain how the study size was arrived at | Methods, paragraph 2 | Of 10,030 participants, we excluded 376 participants with implausibly low or high total energy intake (<500 kcal or >5,000 kcal), 489 participants with cardiovascular disease (myocardial infarction, stroke, or angina) or cancer because diagnosis of chronic diseases may prompt individuals to change their dietary behaviors. We then excluded 3,312 participants who had MetS at baseline. Lastly, we excluded 207 participants with missing covariates. Our final analytic sample was 5,646. |

Continued on next page

| Quantitative variables | 11 | Explain how quantitative variables were handled in the analyses. If applicable, describe which groupings were chosen and why | Methods, paragraph 6 | After summing the scores across each food group, we divided the overall index scores into quintiles for analyses to reflect the design of the plant-based diet indices, and to be consistent with prior studies [12,28]. |
| --- | --- | --- | --- | --- |
| Statistical methods | 12 | (*a*) Describe all statistical methods, including those used to control for confounding | Methods, paragraph 12 | In model 1, we adjusted for age, sex, and total energy intake. In model 2, we additionally adjusted for total energy intake, education, physical activity, smoking status, and alcohol intake. In model 3, we additionally adjusted for BMI. Linear trends were tested by using the median score within each quintile. … |
|  |  | (*b*) Describe any methods used to examine subgroups and interactions |  |  |
|  |  | (*c*) Explain how missing data were addressed | Methods, paragraph 2 | Lastly, we excluded 207 participants with missing covariates. |
|  |  | (*d*) *Cohort study*—If applicable, explain how loss to follow-up was addressed  *Case-control study*—If applicable, explain how matching of cases and controls was addressed  *Cross-sectional study*—If applicable, describe analytical methods taking account of sampling strategy |  |  |
|  |  | (*e*) Describe any sensitivity analyses | Methods, paragraph 9 | Follow-up period was calculated as the time from baseline examination until the date of MetS event or censoring. We defined censoring as participants who did not return for follow-up visit. Participants may not have returned for follow-up visit due to death, but data on vital status were not available in this data set. |
| Results | | | | |
| Participants | 13* | (a) Report numbers of individuals at each stage of study—eg numbers potentially eligible, examined for eligibility, confirmed eligible, included in the study, completing follow-up, and analysed | Methods, paragraph 2 | Of 10,030 participants, we excluded 376 participants with implausibly low or high total energy intake (<500 kcal or >5,000 kcal), 489 participants with cardiovascular disease (myocardial infarction, stroke, or angina) or cancer because diagnosis of chronic diseases may prompt individuals to change their dietary behaviors. We then excluded 3,312 participants who had MetS at baseline. Lastly, we excluded 207 participants with missing covariates. Our final analytic sample was 5,646. |
|  |  | (b) Give reasons for non-participation at each stage |  |  |
|  |  | (c) Consider use of a flow diagram |  |  |
| Descriptive data | 14* | (a) Give characteristics of study participants (eg demographic, clinical, social) and information on exposures and potential confounders | Results, paragraph 1 | Those in the highest quintiles of PDI, hPDI, and provegetarian diet index were more likely to be women, never smokers, more physically active, and consume lower amounts of alcohol. In contrast, those in the highest quintile of uPDI were more likely to be men, less physically active, consumer higher amounts of alcohol; and less likely to be never smokers. Trends in other demographic characteristics (i.e., education), and systolic blood pressure were similar for all diet indices. |
|  |  | (b) Indicate number of participants with missing data for each variable of interest | Methods, paragraph 2 | Lastly, we excluded 207 participants with missing covariates. |
|  |  | (c) *Cohort study*—Summarise follow-up time (eg, average and total amount) | Results, paragraph 2  Table 2 | Over a median follow-up of 8 years,… |
| Outcome data | 15* | *Cohort study*—Report numbers of outcome events or summary measures over time | Results, paragraph 2 | Over a median follow-up of 8 years, 2,583 (45.7%) participants developed incident MetS. |
|  |  | *Case-control study—*Report numbers in each exposure category, or summary measures of exposure |  |  |
|  |  | *Cross-sectional study—*Report numbers of outcome events or summary measures |  |  |
| Main results | 16 | (*a*) Give unadjusted estimates and, if applicable, confounder-adjusted estimates and their precision (eg, 95% confidence interval). Make clear which confounders were adjusted for and why they were included | Results, paragraph 2-3  Table 2, Table 3, Figure 1, S1 Figure | Over a median follow-up of 8 years, 2,583 (45.7%) participants developed incident MetS. There was a strong linear association between higher uPDI score and incident MetS (Fig 1). In model 2, those in the highest quintile of uPDI had 50% (hazard ratio [HR]: 1.50, 95% CI: 1.31, 1.71, P-trend<0.001) higher risk of developing incident MetS compared to those in the lowest quintile of uPDI when we adjusted for demographic characteristics and lifestyle factors (Table 2). |
|  |  | (*b*) Report category boundaries when continuous variables were categorized | Table 1 |  |
|  |  | (*c*) If relevant, consider translating estimates of relative risk into absolute risk for a meaningful time period |  |  |

Continued on next page

| Other analyses | 17 | Report other analyses done—eg analyses of subgroups and interactions, and sensitivity analyses | Results, paragraph 5 | Sensitivity analyses: We found that the associations did not change for PDI (HR for PDIquintile 5 vs. quintile 1: 0.97, 95% CI: 0.86, 1.09, P-trend=0.12),… |
| --- | --- | --- | --- | --- |
| Discussion | | | | |
| Key results | 18 | Summarise key results with reference to study objectives | Discussion, paragraph 1 | In a community-based cohort of South Korean adults, greater adherence to unhealthy plant-based diets (diets high in refined carbohydrates, sugars, salted vegetables, and low in healthy plant foods and animal foods) was associated with a higher risk of incident MetS,… |
| Limitations | 19 | Discuss limitations of the study, taking into account sources of potential bias or imprecision. Discuss both direction and magnitude of any potential bias | Discussion, paragraph 7 | However, several limitations need to be taken into account…. |
| Interpretation | 20 | Give a cautious overall interpretation of results considering objectives, limitations, multiplicity of analyses, results from similar studies, and other relevant evidence | Discussion, paragraph 2-6; paragraph 8 | Our findings on uPDI and incident MetS are generally in agreement with prior studies conducted in Western populations. …  The dietary guidelines for Koreans have recommended eating a balanced diet including a variety of foods such as grains, vegetables, fruits, beans, fish, eggs, meat, poultry, and dairy products; consuming less salt-preserved foods; using less salt when preparing foods; and selecting foods lower in salt, sugar, and fat [45]…. |
| Generalisability | 21 | Discuss the generalisability (external validity) of the study results | Discussion paragraph 7 | Strengths of our study include the use of data from a community-based cohort, validated food frequency questionnaire, repeated dietary assessments, and sufficient follow-up period to ascertain incident MetS. |
| Other information | |  | | |
| Funding | 22 | Give the source of funding and the role of the funders for the present study and, if applicable, for the original study on which the present article is based | Funding section | This work was supported by NRF2018R1D1A1B07045558). The funders had no role in study design, data collection and analysis, or decision to submit the manuscript. |

*Give information separately for cases and controls in case-control studies and, if applicable, for exposed and unexposed groups in cohort and cross-sectional studies.

**Note:** An Explanation and Elaboration article discusses each checklist item and gives methodological background and published examples of transparent reporting. The STROBE checklist is best used in conjunction with this article (freely available on the Web sites of PLoS Medicine at http://www.plosmedicine.org/, Annals of Internal Medicine at http://www.annals.org/, and Epidemiology at http://www.epidem.com/). Information on the STROBE Initiative is available at www.strobe-statement.org.
