# Supplementary material for: Plant-based diets and incident metabolic syndrome: Results from a South Korean prospective cohort study
Source: PLoS Med. 2020 Nov 18;17(11):e1003371. doi: 10.1371/journal.pmed.1003371 (PMC7673569; doi:10.1371/journal.pmed.1003371)
Supplement: S1 Fig — The histogram in gray shows the distribution of plant-based diet scores. The solid lines represent the adjusted HRs for incident MetS, modeled using restricted cubic splines with 4 knots (5th, 35th, 65th, 95th percentiles). The reference point was set at the 5th percentile of each score. The dashed lines represent 95% confidence intervals. HRs were adjusted for age, sex, total energy intake, education, physical activity, smoking status, alcohol intake, and BMI. (DOCX) [file pmed.1003371.s002.docx]

S1 Fig. Adjusted hazard ratios and 95% confidence intervals for incident metabolic syndrome according to the continuous overall plant-based diet index, healthful plant-based diet index, and pro-vegetarian diet index using restricted cubic splines with 4 knots at the 5th, 35th, 65th, and 95th percentiles

| Overall plant-based diet index | Healthful plant-based diet index |
| --- | --- |
| 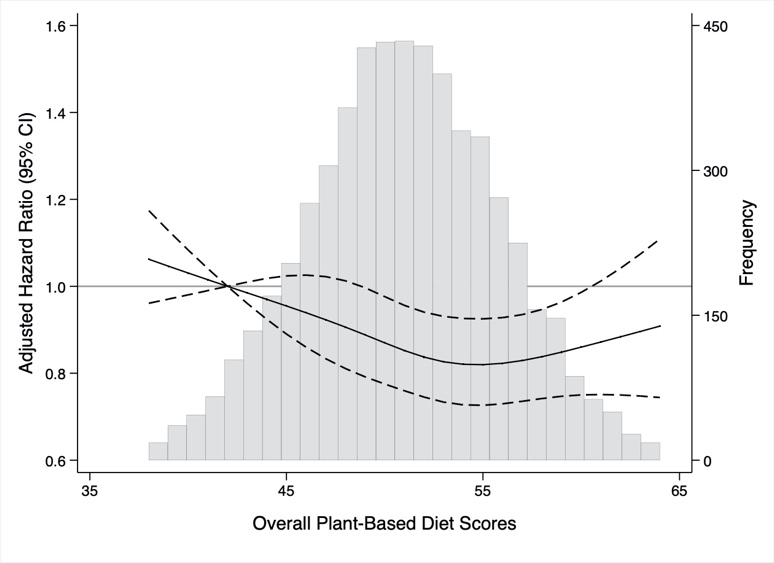 | 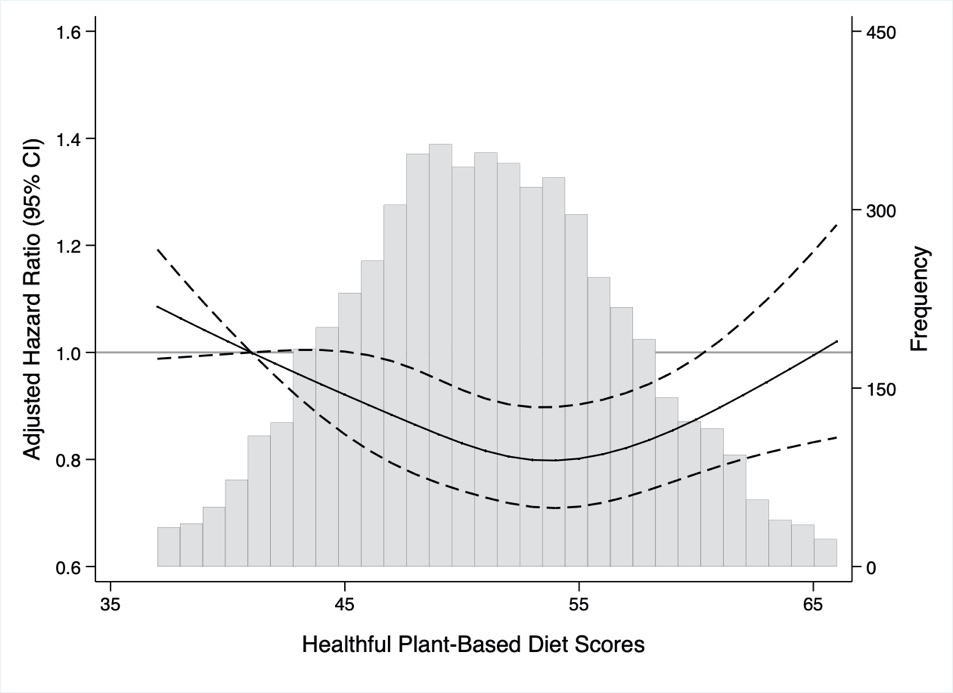 |
| Pro-vegetarian diet index | |
| 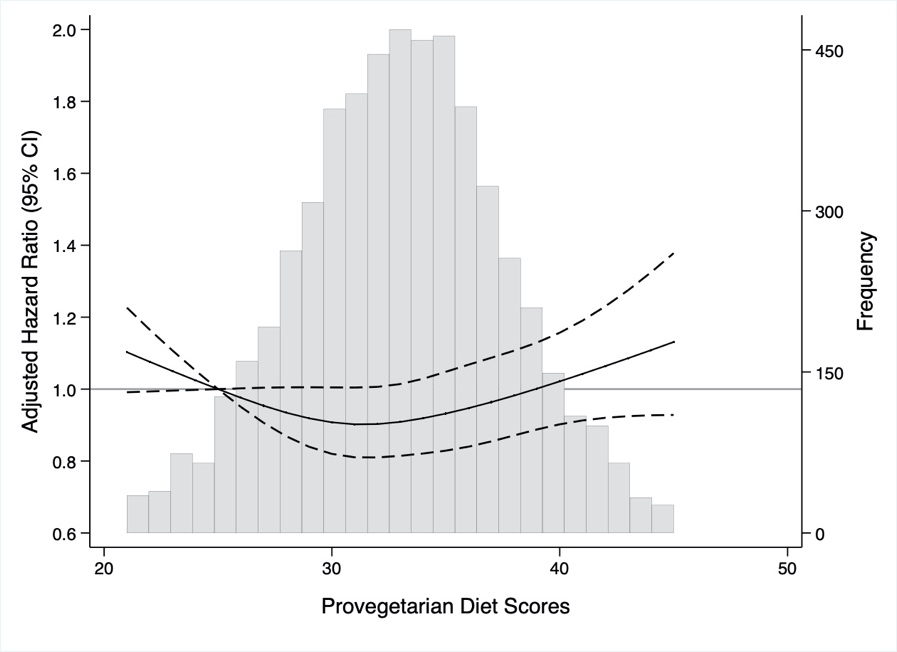 | |

The histogram in gray shows the distribution of plant-based diet scores. The solid lines represent the adjusted hazard ratios for incident MetS, modeled using restricted cubic splines with 4 knots (5^th^, 35^th^, 65^th^, 95^th^ percentiles). The reference was point was set at the 5^th^ percentile of each score. The dashed lines represent 95% confidence intervals. Hazard ratios were adjusted for age, sex, total energy intake, education, physical activity, smoking status, alcohol intake, and body mass index.
