# Supplementary material for: Plant-based diets and incident metabolic syndrome: Results from a South Korean prospective cohort study
Source: PLoS Med. 2020 Nov 18;17(11):e1003371. doi: 10.1371/journal.pmed.1003371 (PMC7673569; doi:10.1371/journal.pmed.1003371)
Supplement: S1 Table — 1The PDI, hPDI, and uPDI categorized food groups into “healthy plant foods,” “less healthy plant foods,” and “animal foods.” The pro-vegetarian diet index categorized food groups into “plant foods” and “animal foods.” Positive indicates that higher intakes received higher scores. Reverse indicates that higher intakes received lower scores. 2 Whole grains and refined grains were aggregated to a “grains” food group in the pro-vegetarian diet index. hPDI, healthful plant-based diet index; PDI, overall plant-based diet index; uPDI, unhealthful plant-based diet index (DOCX) [file pmed.1003371.s003.docx]

S1 Table. Scoring system and classification of food items in the Korean Genome and Epidemiology Study (KoGES)^1^

| Food groups | Items in the food frequency questionnaire | PDI | hPDI | uPDI | Pro-vegetarian |
| --- | --- | --- | --- | --- | --- |
| **Healthy plant foods** | | | | | |
| Whole grains | Mixed grains, barley, grain with beans | Positive | Positive | Reverse | Positive^2^ |
| Fruits | Strawberry, watermelon, banana, peach/ plum, oriental melon/melon, persimmon/dried persimmon, pear/pear juice, tangerine, orange/orange juice, apple/apple juice, grape/grape juice | Positive | Positive | Reverse | Positive |
| Vegetables | Sweet potatoes, radish, napa cabbage/napa cabbage soup, spinach, lettuce, perilla leaves, sesame leaves/vegetable salad, other green vegetable, Deodeok/bellflower root, bean sprouts/mung-bean sprouts, bracken/sweet potato stem, oyster mushroom, other mushrooms, green pepper leaf/chamnamul, crown daisy /chive /watercress, cucumber, carrot/carrot juice, onion, green peppers, zucchini, pumpkin/kabocha squash, laver, kelp/seaweed, tomato/tomato juice | Positive | Positive | Reverse | Positive |
| Nuts | Peanuts/almonds/pine nuts | Positive | Positive | Reverse | Positive |
| Legumes | Beans/beans cooked in soy sauce, tofu, bean curd, soybean milk | Positive | Positive | Reverse | Positive |
| Tea and coffee | Coffee, green tea | Positive | Positive | Reverse | Not scored |
| **Less healthy plant foods** | | | | | |
| Refined grains | White rice, instant noodles, other noodles (udon noodles), black bean sauce noodles, cold noodles, rice cake/rice cake soup, other rice cakes, cereals, white breads, other breads, grain powder, starch jelly, stir-fried noodles and vegetables | Positive | Reverse | Positive | Positive^2^ |
| Potatoes | Potatoes | Positive | Reverse | Positive | Positive |
| Sugar sweetened beverages | soft drink, other beverages (sweetened rice tea, citron tea) | Positive | Reverse | Positive | Not scored |
| Sweets and desserts | Sweet red bean bread, cake/chocolate pie, cookies/crackers/snacks, candies/chocolates, sugars (added to tea or coffee) | Positive | Reverse | Positive | Not scored |
| Salty food group | Bean paste, Bean paste/bean paste soup, Kimchi (Korean cabbage, radish), watery radish kimchi, other kimchi, pickled vegetable (preserved in soy sauce or salt), radish kimchi (preserved in soy sauce or salt) | Positive | Reverse | Positive | Not scored |
| **Animal foods** | | | | | |
| Animal fat | Butter, cream (added to tea or coffee) | Reverse | Reverse | Reverse | Reverse |
| Dairy | Milk, yogurt/yoplait, ice cream, cheese | Reverse | Reverse | Reverse | Reverse |
| Eggs | Eggs/quail eggs | Reverse | Reverse | Reverse | Reverse |
| Fish | Sashimi, belt fish, mackrele/pacific saury, eel, yellow croaker/sea bream/sole, alaska pollack/frozen pollack/dried pollack, squid/dried squid/octopus, anchovy/stir-fried anchovy, canned tuna, salted shrimp/salted fish, clam/sea snail, oyster, crab/marinated crab, shrimp, fishcake | Reverse | Reverse | Reverse | Reverse |
| Meat | Pork belly, grilled pork/stir-fried pork/pork bulgogi/Korean meatball, steamed pork, processed meat (ham,sausage), organ meat/Korean sausage, steak/grilled beef, dog meat, beef soup, chicken(fried, stew, braised spicy chicken), beef stew | Reverse | Reverse | Reverse | Reverse |
| Miscellaneous animal foods | dumplings/dumpling soup, pizza/hamburger | Reverse | Reverse | Reverse | Not scored |

^1^ The PDI, hPDI, and uPDI categorized good groups to “healthy plant foods,” “less healthy plant foods,” and “animal foods.” The pro-vegetarian diet index categorized food groups into “plant foods” and “animal foods.” Positive indicates that higher intakes received higher scores. Reverse indicates that higher intakes received lower scores.

^2^ Whole grains and refined grains were aggregated to “grains” food group in the pro-vegetarian diet index.

PDI, overall plant-based diet index; hPDI, healthful plant-based diet index; uPDI, unhealthful plant-based diet index
