# Supplementary material for: Plant-based diets and incident metabolic syndrome: Results from a South Korean prospective cohort study
Source: PLoS Med. 2020 Nov 18;17(11):e1003371. doi: 10.1371/journal.pmed.1003371 (PMC7673569; doi:10.1371/journal.pmed.1003371)
Supplement: S2 Table — (DOCX) [file pmed.1003371.s004.docx]

S2 Table. Nutritional characteristics of diet according to quintiles of plant-based diet indices

|  | Quintile 1 | Quintile 2 | Quintile 3 | Quintile 4 | Quintile 5 | *P*-value |
| --- | --- | --- | --- | --- | --- | --- |
| **Overall plant-based diet index** | | | | | | |
| Total Energy Intake, kcal/d | 2020.2 (549.9) | 1872.4 (521.1) | 1802.7 (480.5) | 1831.1 (534.9) | 1922.8 (513.9) | <0.001 |
| Carbohydrate, % of energy | 68.2 (6.0) | 70.5 (6.2) | 71.9 (5.9) | 72.3 (5.5) | 73.8 (5.0) | <0.001 |
| Protein, % of energy | 13.8 (2.0) | 13.4 (2.1) | 13.1 (2.1) | 13.2 (2.1) | 12.9 (1.8) | <0.001 |
| Fat, % of energy | 12.9 (3.7) | 11.6 (3.8) | 10.7 (3.6) | 10.5 (3.3) | 9.8 (3.1) | <0.001 |
| Calcium, mg/1000 kcal | 238.0 (84.0) | 242.4 (89.3) | 235.0 (87.7) | 246.3 (86.5) | 247.0 (78.6) | 0.004 |
| Phosphorus, mg/1000 kcal | 514.2 (75.0) | 515.5 (80.1) | 510.2 (82.0) | 520.1 (79.7) | 515.6 (70.9) | 0.084 |
| Iron, mg/1000 kcal | 5.1 (1.1) | 5.4 (1.2) | 5.4 (1.3) | 5.7 (1.3) | 6.0 (1.3) | <0.001 |
| Potassium, mg/1000 kcal | 1163.1 (281.0) | 1241.8 (297.0) | 1284.1 (331.7) | 1361.6 (324.0) | 1424.7 (303.5) | <0.001 |
| Sodium, mg/1000 kcal | 1369.1 (480.4) | 1527.3 (577.4) | 1643.9 (640.8) | 1753.6 (654.8) | 1847.1 (627.4) | <0.001 |
| Niacin, mg/1000 kcal | 7.9 (1.4) | 7.8 (1.4) | 7.9 (1.5) | 8.0 (1.4) | 8.0 (1.3) | 0.004 |
| Vitamin C, mg/1000 kcal | 50.9 (26.7) | 57.5 (26.4) | 62.2 (30.5) | 67.0 (28.4) | 74.7 (29.8) | <0.001 |
| Zinc, mg /1000 kcal | 4.6 (1.0) | 4.4 (0.8) | 4.3 (0.8) | 4.4 (0.9) | 4.3 (0.9) | <0.001 |
| Vitamin B-6, mg/1000 kcal | 0.9 (0.1) | 0.9 (0.2) | 0.9 (0.2) | 0.9 (0.2) | 1.0 (0.2) | <0.001 |
| Folate, μg/1000 kcal | 106.4 (31.3) | 118.4 (35.1) | 123.7 (38.5) | 133.2 (39.0) | 144.1 (40.6) | <0.001 |
| Beta-carotene, μg/1000 kcal | 1105.9 (612.3) | 1287.0 (766.6) | 1368.6 (781.7) | 1459.8 (774.9) | 1659.5 (890.8) | <0.001 |
| Fiber, g/1000 kcal | 2.9 (0.8) | 3.3 (0.9) | 3.5 (1.0) | 3.8 (1.0) | 4.2 (1.0) | <0.001 |
| Vitamin E, mg/1000 kcal | 4.4 (1.2) | 4.5 (1.2) | 4.6 (1.3) | 4.8 (1.3) | 5.0 (1.3) | <0.001 |
| Cholesterol, mg/1000 kcal | 106.8 (44.2) | 90.4 (43.4) | 82.7 (43.7) | 78.0 (41.0) | 65.8 (36.5) | <0.001 |
| **Healthful plant-based diet index** | | | | | | |
| Total Energy Intake, kcal/d | 1915.5 (543.4) | 1798.1 (537.5) | 1835.7 (505.7) | 1925.5 (504.1) | 2034.6 (521.6) | <0.001 |
| Carbohydrate, % of energy | 68.7 (5.9) | 70.8 (6.4) | 71.4 (5.8) | 72.2 (5.6) | 73.4 (5.5) | <0.001 |
| Protein, % of energy | 13.8 (2.0) | 13.3 (2.3) | 13.3 (2.1) | 13.2 (2.0) | 13.1 (1.9) | <0.001 |
| Fat, % of energy | 12.8 (3.6) | 11.5 (3.9) | 11.1 (3.5) | 10.6 (3.4) | 9.8 (3.3) | <0.001 |
| Calcium, mg/1000 kcal | 249.1 (79.7) | 241.4 (91.1) | 240.4 (86.4) | 242.0 (83.7) | 234.8 (85.8) | 0.002 |
| Phosphorus, mg/1000 kcal | 515.1 (72.1) | 508.2 (84.3) | 512.8 (79.0) | 520.2 (77.5) | 521.3 (74.4) | <0.001 |
| Iron, mg/1000 kcal | 5.2 (1.2) | 5.3 (1.3) | 5.5 (1.2) | 5.7 (1.2) | 5.9 (1.3) | <0.001 |
| Potassium, mg/1000 kcal | 1261.7 (286.0) | 1263.9 (324.9) | 1285.3 (314.7) | 1303.0 (334.0) | 1329.7 (342.6) | <0.001 |
| Sodium, mg/1000 kcal | 1766.2 (636.2) | 1654.1 (620.0) | 1598.2 (607.7) | 1512.4 (580.5) | 1473.5 (580.4) | <0.001 |
| Niacin, mg/1000 kcal | 8.2 (1.4) | 7.9 (1.5) | 7.9 (1.4) | 7.8 (1.4) | 7.7 (1.3) | <0.001 |
| Vitamin C, mg/1000 kcal | 56.5 (23.1) | 59.0 (26.5) | 62.3 (28.1) | 63.9 (32.5) | 68.4 (35.9) | <0.001 |
| Zinc, mg /1000 kcal | 4.5 (1.0) | 4.4 (1.1) | 4.4 (0.9) | 4.4 (0.8) | 4.4 (0.8) | 0.024 |
| Vitamin B-6, mg/1000 kcal | 0.9 (0.2) | 0.9 (0.2) | 0.9 (0.2) | 0.9 (0.2) | 0.9 (0.2) | 0.20 |
| Folate, μg/1000 kcal | 119.5 (36.7) | 120.0 (38.5) | 123.9 (37.8) | 125.9 (38.9) | 132.2 (42.3) | <0.001 |
| Beta-carotene, μg/1000 kcal | 1352.4 (741.4) | 1326.4 (722.2) | 1371.2 (799.3) | 1340.5 (783.6) | 1401.6 (873.9) | 0.21 |
| Fiber, g/1000 kcal | 3.2 (0.9) | 3.3 (1.0) | 3.5 (1.0) | 3.6 (1.0) | 3.9 (1.1) | <0.001 |
| Vitamin E, mg/1000 kcal | 4.7 (1.2) | 4.5 (1.3) | 4.6 (1.3) | 4.6 (1.3) | 4.7 (1.4) | 0.018 |
| Cholesterol, mg/1000 kcal | 105.5 (43.1) | 88.8 (45.7) | 85.0 (43.2) | 78.2 (39.9) | 67.3 (38.9) | <0.001 |
| **Unhealthful plant-based diet index** | | | | | | |
| Total Energy Intake, kcal/d | 1821.2 (493.9) | 1860.9 (512.0) | 1852.1 (475.0) | 1916.0 (539.5) | 2085.2 (596.8) | <0.001 |
| Carbohydrate, % of energy | 67.9 (5.4) | 70.2 (5.5) | 71.2 (5.8) | 72.8 (5.9) | 74.5 (5.7) | <0.001 |
| Protein, % of energy | 14.8 (1.9) | 13.8 (1.8) | 13.3 (1.9) | 12.6 (1.8) | 11.9 (1.7) | <0.001 |
| Fat, % of energy | 13.0 (3.3) | 11.7 (3.4) | 11.1 (3.5) | 10.3 (3.7) | 9.6 (3.6) | <0.001 |
| Calcium, mg/1000 kcal | 291.7 (88.4) | 258.3 (81.8) | 234.1 (75.8) | 215.3 (73.1) | 196.0 (68.5) | <0.001 |
| Phosphorus, mg/1000 kcal | 575.6 (72.4) | 535.7 (65.6) | 509.5 (64.6) | 484.7 (62.0) | 453.4 (56.9) | <0.001 |
| Iron, mg/1000 kcal | 6.3 (1.1) | 5.8 (1.2) | 5.4 (1.1) | 5.1 (1.1) | 4.7 (1.1) | <0.001 |
| Potassium, mg/1000 kcal | 1477.1 (313.0) | 1349.2 (298.9) | 1251.3 (280.2) | 1193.2 (287.4) | 1114.0 (281.1) | <0.001 |
| Sodium, mg/1000 kcal | 1565.5 (583.9) | 1589.0 (592.3) | 1599.6 (605.4) | 1644.2 (633.4) | 1675.0 (671.8) | <0.001 |
| Niacin, mg/1000 kcal | 8.7 (1.4) | 8.2 (1.3) | 7.9 (1.3) | 7.6 (1.2) | 7.0 (1.2) | <0.001 |
| Vitamin C, mg/1000 kcal | 70.2 (30.4) | 64.6 (29.1) | 58.7 (27.3) | 57.8 (29.6) | 55.3 (28.0) | <0.001 |
| Zinc, mg /1000 kcal | 4.8 (1.0) | 4.5 (0.8) | 4.4 (0.9) | 4.2 (0.9) | 4.0 (0.8) | <0.001 |
| Vitamin B-6, mg/1000 kcal | 1.0 (0.2) | 0.9 (0.2) | 0.9 (0.2) | 0.9 (0.2) | 0.8 (0.2) | <0.001 |
| Folate, μg/1000 kcal | 136.4 (36.5) | 129.1 (39.1) | 120.7 (35.8) | 118.5 (39.9) | 112.6 (39.8) | <0.001 |
| Beta-carotene, μg/1000 kcal | 1464.2 (753.2) | 1411.1 (823.4) | 1329.9 (759.3) | 1307.2 (818.0) | 1260.3 (764.8) | <0.001 |
| Fiber, g/1000 kcal | 3.7 (1.0) | 3.6 (1.0) | 3.4 (1.0) | 3.4 (1.1) | 3.3 (1.0) | <0.001 |
| Vitamin E, mg/1000 kcal | 5.3 (1.2) | 4.8 (1.2) | 4.5 (1.2) | 4.3 (1.2) | 4.1 (1.3) | <0.001 |
| Cholesterol, mg/1000 kcal | 113.2 (42.7) | 94.0 (40.5) | 84.1 (41.5) | 72.4 (39.7) | 59.2 (35.3) | <0.001 |
| **Pro-vegetarian diet index** | | | | | | |
| Total Energy Intake, kcal/d | 2052.4 (529.1) | 1839.1 (513.1) | 1818.2 (508.4) | 1830.5 (507.3) | 1936.5 (550.0) | <0.001 |
| Carbohydrate, % of energy | 66.9 (5.5) | 70.1 (5.7) | 71.7 (5.4) | 73.3 (5.2) | 74.8 (5.1) | <0.001 |
| Protein, % of energy | 14.2 (2.0) | 13.5 (2.2) | 13.2 (2.0) | 12.9 (2.0) | 12.6 (1.7) | <0.001 |
| Fat, % of energy | 13.8 (3.3) | 11.9 (3.4) | 10.9 (3.3) | 9.8 (3.1) | 9.0 (3.1) | <0.001 |
| Calcium, mg/1000 kcal | 254.1 (80.9) | 246.7 (94.3) | 241.7 (85.7) | 235.2 (85.9) | 227.5 (74.5) | <0.001 |
| Phosphorus, mg/1000 kcal | 528.8 (70.9) | 518.0 (84.8) | 514.5 (79.7) | 507.8 (79.0) | 503.5 (68.3) | <0.001 |
| Iron, mg/1000 kcal | 5.3 (1.1) | 5.4 (1.3) | 5.5 (1.3) | 5.5 (1.3) | 5.8 (1.2) | <0.001 |
| Potassium, mg/1000 kcal | 1265.3 (273.7) | 1276.1 (343.4) | 1296.6 (321.5) | 1282.6 (337.4) | 1326.5 (317.1) | <0.001 |
| Sodium, mg/1000 kcal | 1548.1 (541.9) | 1599.3 (652.0) | 1629.3 (609.9) | 1635.4 (653.2) | 1663.7 (618.2) | <0.001 |
| Niacin, mg/1000 kcal | 8.4 (1.4) | 8.0 (1.5) | 7.9 (1.4) | 7.6 (1.3) | 7.6 (1.2) | <0.001 |
| Vitamin C, mg/1000 kcal | 54.3 (24.5) | 58.4 (29.5) | 62.8 (28.7) | 64.0 (29.7) | 72.0 (32.1) | <0.001 |
| Zinc, mg /1000 kcal | 4.6 (1.1) | 4.4 (0.9) | 4.4 (1.0) | 4.3 (0.8) | 4.2 (0.8) | <0.001 |
| Vitamin B-6, mg/1000 kcal | 0.9 (0.1) | 0.9 (0.2) | 0.9 (0.2) | 0.9 (0.2) | 0.9 (0.2) | <0.001 |
| Folate, μg/1000 kcal | 113.5 (33.4) | 120.2 (40.2) | 125.2 (36.6) | 126.8 (39.0) | 138.7 (41.4) | <0.001 |
| Beta-carotene, μg/1000 kcal | 1249.3 (707.3) | 1307.7 (781.9) | 1357.9 (749.5) | 1380.7 (764.3) | 1549.8 (904.0) | <0.001 |
| Fiber, g/1000 kcal | 3.0 (0.8) | 3.3 (1.0) | 3.5 (0.9) | 3.7 (1.0) | 4.1 (1.0) | <0.001 |
| Vitamin E, mg/1000 kcal | 4.6 (1.1) | 4.6 (1.4) | 4.6 (1.3) | 4.6 (1.3) | 4.8 (1.4) | 0.002 |
| Cholesterol, mg/1000 kcal | 114.5 (42.6) | 92.1 (42.2) | 83.2 (40.4) | 73.0 (39.3) | 59.6 (34.3) | <0.001 |
