# Supplementary material for: Plant-based diets and incident metabolic syndrome: Results from a South Korean prospective cohort study
Source: PLoS Med. 2020 Nov 18;17(11):e1003371. doi: 10.1371/journal.pmed.1003371 (PMC7673569; doi:10.1371/journal.pmed.1003371)
Supplement: S1 Text — (PDF) [file pmed.1003371.s005.pdf]

Research Plan

1. Summary

1.1 Research Information						
**1.1.1 Study title**	**Korean**	대규모 인구집단 조사 자료를 활용한 한국인의 대사성질환과 관련된 식이 위험 요인 규명				
	**English**	Dietary factors associated with cardiometabolic diseases in Korean adults using a large population study				
**1.1.2 Principle Investigator**	**Institute**	**Position**	**ID**	**Name**		
	Graduate School of East-West Medical Science	professor	026105	Jihye Kim		
	**Contact**		**E-mail**			
	031-201-3497		kjhye@khu.ac.kr			
**1.1.3 Collaborators**	**Name**	**Institute**	**Position**	**Contact**	**e-mail address**	**Role**
	Hyunju Kim	Johns Hopkins Bloomberg School of Public Health	Postdoctoral fellow		hkim25@jhu.edu	Data analysis
	Kyueun Lee	Graduate School of East-West Medical Science, Kyung Hee University	Master's student	031-201-2369	kyueun07@khu.ac.kr	Data analysis

1.2 Research Information	
**1.2.1 Study design**	√Human (□ Survey √Observational study □ Behavior research □ Clinical trial □ Education program □ Sensory test)<br>☐ Human origin sample (□ secondary sample analysis □ New sampling)<br>☐ Genetic study<br>☐ Privacy<br>☐ Others ( )
**1.2.2 Study objective**	This study explores the relationship between dietary factors (food or food group, dietary pattern, diet quality) and metabolic syndrome and its related chronic diseases such as obesity, hypertension, diabetes, cardiovascular disease, and cancer in Korean adults.
**1.2.3 Study period**	One year after IRB permission

**1.2.4 Study location**	Research Institute: Department of Medical Nutrition, Graduate School of East-West Medical Science, Kyung Hee University<br>Address: 1732 Deogyeong-daero, Giheung-gu, Yongin, Gyeonggi-do, 17104

We submit this research plan to seek approval from the Institutional Review Board.

Date: December/9/2019

Principle investigator: Jihye Kim (signature)

2. Research Plan

2.1 Research Information

2.1.1 Grant source

Not applicable

2.1.2 Objective of study

This study explores the relationship between dietary factors (food or food group, dietary pattern, diet quality) and metabolic syndrome and its related chronic diseases such as obesity, hypertension, diabetes, cardiovascular disease, and cancer in Korean adults.

2.1.3 Background

❍ Incidence of metabolic syndrome among Korean population

- Metabolic syndrome (MetS) is a cluster of conditions [abdominal obesity, high blood glucose, hypertriglyceridemia, low HDL cholesterol (HDL-C), and elevated blood pressure] that is strongly associated with development of cardiovascular disease (Nutr Metab Cardio vasc Dis 2010; Diabetes Care 2005).
- The prevalence of MetS has increased around the world and individuals with metabolic syndrome had a 2.2 fold higher risk for cardiovascular disease and a 3 fold higher risk for type 2 diabetes (J Am Coll Cardiol 2007; Diabetes Care 2007).
- The prevalence of MetS has rapidly increasing in Korean adults as well as children and adolescents (Diabetes Care 2007; Ministry of Health and Welfare 2005) According to the Korea National Health and Nutrition Examination Survey III, the prevalence of MetS was 33.1 % for men and 26% for women among Korean adults aged 30 years old and more (Ministry of Health and Welfare 2005).

❍ Dietary factors related to metabolic syndrome

- In previous studies, metabolic syndrome is known to be closely related to dietary intake. Recently, it has been known that it is closely related to food, food group intake, and dietary pattern.
- Whole grains : In a cross-sectional study of adults over the age of 18, reported that upper category of whole grain intake had 39% lower prevalence of hypertriglyceridemia, 16% lower prevalence of hypertension and 32% lower prevalence of metabolic syndrome than lowest category of whole grain intake. Higher consumption of refined grains was associated with 23% higher prevalence of having hypercholestrolemia, 2 fold higher prevalence of hypertriglyceridemia, 69% higher prevalence of hypertension and 2.2 fold higher prevalence of metabolic syndrome than lowest category of refined grain intake (Eur J Clin Nutr 2005).
- Legume: Subjects in the highest quartile of legume intake had 75% lower prevalence of having metabolic syndrome compared with those in the lowest quartile of legume intake. Subjects in the highest versus lowest quartile of legume intake have significantly reduced risk of metabolic syndrome (Arch Iran Med 2012).
- Fish: A cross-sectional study of Iranian women reported that subjects in the highest tertile of fish intake were 65% less likely to have the metabolic syndrome than those in the lowest tertile (Eur J Clin Nutr 2014).
- Red meat: In a study of Iranian women between the ages of 40 and 60, individuals in the top quintile of red meat intake had 2.3 fold greater prevalence of having metabolic syndrome compared with those in the bottom quintile (J Nutr 2009).
- Milk and dairy product: In the French general population, higher total dairy and cheese intake were associated with decreased waist circumference and triglycerides during the 9-year follow-up (J Am Coll Nutr 2011). Using data from the fifth Korean National Health and Nutrition Examination Survey, researchers found that higher consumption of milk or yogurt was significantly associated with a 29% lower

risk of the metabolic syndrome (J Hum Nutr Diet 2013). A recent cohort study, which conducted in 26,445 healthy men and women followed up during 12 years, reported that highest versus lowest intake category of fermented milk was associated with 15% decreased incidence of cardiovascular disease (Eur J Epidemiol 2011).

- Fruit and vegetable: In a study of adults between the ages of 40 and 60, reported subjects in the highest quintile of fruit intake had a 34% lower and those in the highest quintile of vegetables intake had a 30% lower chance of having the metabolic syndrome than did those in the lowest quintiles (Am J Clin Nutr 2006).
- Sugar sweetened beverage: In precious cross sectional study, which was conducted tin Mexican aged between 20 and 70 years reported that subjects consuming more than two servings of sweetened beverages daily were having 2 times greater risk of metabolic syndrome than those who did not consume sweetened beverages (Public Health Nutr 2010).
- Alcohol: The cohort study of Italian men whose aged over 65 years reported that high alcohol intake significantly increases the risk of developing metabolic syndrome compared to those who consume low alcohol intake (Eur J Clin Nutr 2010).
- Refined grain: Data from the Fourth Korea National Health and Nutrition Examination Survey reported that women in the highest quintiles of refined grains and white rice intakes were more likely to have metabolic syndrome than women in the lowest quintiles (J Acad Nutr Diet 2014).
- Dietary pattern and diet quality: Recent studies have shown that dietary patterns composed of healthy foods such as Mediterranean diets and DASH diet reduced the risk of developing metabolic syndrome, diabetes, high blood pressure and cardiovascular disease. Additionally, previous studies reported that indicators such as healthy eating index, alternative health eating index, inflammation index, were associated with metabolic syndrome, cardiovascular disease, hypertension, diabetes, colon cancer and rectal cancer (BMC Endocrine Disorders 2019, J Hum Nutr Diet 2017, Nutr Cancer 2018, Nutr Metab Cardiovasc Dis 2019, Nutr Cancer 2019, Nutrients 2017)
- Several cross-sectional studies have been conducted between metabolic diseases and dietary risk factors, but most of them are conducted in limited population subgroups. In this study, we aim to provide appropriate dietary guidelines for the prevention and management of metabolic diseases in Koreans using large-scale population-based data (cohort) represented by Koreans. We aim to identify dietary risk factors associated with the development of metabolic diseases because some of these data have been followed up for a long term.

2.1.4 Expected results

❍ Based on the large-scale population survey data, we believe that it is possible to suggest the future direction on related chronic diseases if we identify the associated dietary risk factors in Korean adults such as metabolic syndrome and related metabolic diseases (obesity, high blood pressure, diabetes, cardiovascular disease and cancer).

❍ The findings can be used as a basis for establishing and informing national welfare policies for the implementation of a healthy society.

2.2 Study population

2.2.1 Study population

Approximately, 210,000 participants from the Korean Genome and Epidemiology Study (KoGES) established at Korea Centers for Disease Control and Prevention are included for the analysis (10,030 participants from the KoGES_Ansan and Ansung study, 173,357 participants from the KoGES_HEXA study, 28338 participants from the Koges_CAVAS study)

2.2.2 Criteria for drop out

Not applicable

2.2.3 Criteria for Study Discontinuation
Not applicable

2.2.4 Sample size of study population
This study will identify dietary factors related to metabolic diseases in Korean adults. We made assumptions about diabetes because diabetes has the lowest incidence in this data set. A previous study which showed a 12% reduction in the risk of diabetes among those with higher versus lowest quality of diet reported that the number of samples required at power 80 % was approximately 220,000 (PLoS Med, 2016). The number of participants is similar to the total number of cohort participants of 210,000, requiring 210,000 participants.

2.2.5 Recruitment of study subjects
Not applicable

2.2.6 Consent form for study participation
Not applicable

2.2.7 Strategy for Vulnerable subjects
Not applicable

2.2.8 Reward for study participation
Not applicable

2.2.9 Risk or side effect for study participation
Not applicable

2.2.10 Disadvantage for study discontinuation or refuse of study participation
Not applicable

2.2.11 Privacy policy
Privacy data from cohort research are not provided to the researchers who receive cohort data. The information provided to the researchers will be managed by a unique identification ID and the data obtained from this study will be accessible only to the researchers participating in the study. After the end of the study, the results of this study will not be discarded, but personal identification ID and health-related information will be discarded permanently after the end of the study.

2.3 Study Method
2.3.1 Study period
One year after IRB permission

2.3.2 Study location
Human Nutrition laboratory, Department of Medical Nutrition, Graduate School of East-West Medical Science, Kyung Hee University, Yongin, South Korea

2.3.3 Study design

A prospective cohort study from the Korean Genome and Epidemiology Study (KoGES)_Ansan and Ansung study and a cross-sectional study from the KoGES_HEXA

2.3.4 Schedule for study procedure

Activities	Timeline				Notes
	First quarter	Second quarter	Third quarter	Fourth quarter	
IRB approval, application to receive data, literature review	O				
Investigate the cross-sectional association between dietary factors and metabolic syndrome		O	O		
Investigate the prospective association between dietary factors and metabolic syndrome			O		
Interim report and manuscript writing				O	

2.3.5 (For clinical trial) control, randomization, blind

Not applicable

2.3.6 (For clinical trial) dosage, protocol, intervention period

Not applicable

2.3.7 Methods

❍ Assessment of food and nutrient intake

Dietary pattern and diet quality index will be evaluated as Plant-based diet indices, Mediterranean diet, Healthy eating index, Alternate Healthy Eating Index, Dietary Approaches to Stop Hypertension Score, Inflammatory index (J Acad Nutr Diet 2015, Nutrients 2017)

❍ Ascertainment of metabolic syndrome

Metabolic syndrome is defined based on the criteria established by the National Cholesterol Education Program Adult Treatment Panel III, and modified by the American Heart Association and the National Heart, Lung, and Blood Institute. Incident MetS is defined as having 3 or more of the following conditions: (Circulation, 2009)

- · Abdominal obesity: waist circumference > 90cm (Men) or >80cm (women) (Circulation, 2005)
- · High fasting blood glucose: fasting glucose > 100 mg/dL or doctor's diagnosis of diabetes mellitus, or the use of hypoglycemic drug, insulin administration
- · Hypertriglyceridemia: Triglyceride > 150 mg/dL
- · Low high density lipoprotein–cholesterol (HDL-C): HDL-C<40 mg/dL(men), <50mg/dL (women)
- · Elevated blood pressure: systolic blood pressure / diastolic blood pressure > 130/85 mmHg or antihypertensive medication use

❍ Ascertainment of diabetes: fasting glucose ≥ 126 mg/dL, use of hypoglycemic drug, insulin administration, HbA1c>6.5%

❍ Ascertainment of hypertension: systolic blood pressure>140 mm Hg or diastolic blood pressure >90

mm Hg or antihypertensive medication use

❍ Ascertainment of overweight and obesity: overweight is defined as 23≤body mass index (BMI)<25 and obesity is defined as BMI≥25 kg/m^2

❍ Ascertainment of cancer: Incidence of gastric cancer, colon cancer, breast cancer, and thyroid cancer are examined.

❍ Measurement of biochemical indicators: blood glucose, triglyceride, cholesterol, blood pressure, insulin, and hs-CRP were meausred

❍ Covariates: demographic variables and lifestyle factors such as age, sex, education, smoking status, alcohol drinking, physical activity, nutrient intake will be used as covariates

❍ Anthropometric measurements will be used as covariate to evaluate the relationship between dietary factors and metabolic diseases

2.3.8 Data collection

Not applicable

2.3.9 Statistical analysis

All data are analyzed using SAS version 9.4 (SAS institute, Cary, NC, USA). P values < 0.05 are considered as statistically significant. To evaluate the prospective associations between dietary factors and incidence of metabolic diseases such as metabolic syndrome, hypertension, diabetes, we will use Cox proportional hazards models. To investigate the cross-sectional association, we will use logistic regression analysis.

2.3.10 Concerns about side effects and safety

Not applicable

2.4 Data protection

2.4.1 Data protection

The information provided to the researchers will be managed by a unique identification ID and the data obtained from this study will be accessible only to the researchers participating in the study. After the end of the study, the results of this study will not be discarded, but personal identification ID and health-related information will be discarded permanently after the end of the study.

2.4.2 Compensation policies for side effects and safety

Not applicable

2.5 References

Alberti KG, Eckel RH, Grundy SM, Zimmet PZ, Cleeman JI et al. (2009) “Harmonizing the metabolic syndrome: a joint interim statement of the International Diabetes Federation Task Force on Epidemiology and Prevention; National Heart, Lung, and Blood Institute; American Heart Association; World Heart Federation; International Atherosclerosis Society; and International Association for the Study of Obesity”. Circulation 120:1640-1645.

Ambrosini, G. L., R. C. Huang, T. A. Mori, B. P. Hands, T. A. O'Sullivan, N. H. de Klerk, L. J. Beilin and W. H. Oddy(2010). "Dietary patterns and markers for the metabolic syndrome in Australian adolescents." Nutr Metab Cardio vasc Dis **20**(4): 274-283.

Azadbakht L1, Esmaillzadeh A. (2009) “Red meat intake is associated with metabolic syndrome and the plasma C-reactive protein concentration in women.” J Nutr. 2009 Feb;139(2):335-9.

Buja, A., E. Scafato, G. Sergi, S. Maggi, M. A. Suhad, G. Rausa, A. Coin, I. Baldi, E. Manzato, L. Galluzzo, G. Enzi, E. Perissinotto and I. W. Group (2010). "Alcohol consumption and metabolic syndrome in the elderly: results from the Italian longitudinal study on aging." Eur J Clin Nutr **64**(3): 297-307.

Denova-Gutierrez, E., J. O. Talavera, G. Huitron-Bravo, P. Mendez-Hernandez and J. Salmeron (2010). "Sweetened beverage consumption and increased risk of metabolic syndrome in Mexican adults." Public Health Nutr **13**(6): 835-842.

Esmaillzadeh, A., M. Kimiagar, Y. Mehrabi, L. Azadbakht, F. B. Hu and W. C. Willett (2006). "Fruit and vegetable intakes, C-reactive protein, and the metabolic syndrome." Am J Clin Nutr **84**(6): 1489-1497.

Esmaillzadeh, A., P. Mirmiran and F. Azizi (2005). "Whole-grain consumption and the metabolic syndrome: a favorable association in Tehranian adults." Eur J Clin Nutr **59**(3): 353-362.

Fumeron, F., A. Lamri, N. Emery, N. Bellili, R. Jaziri, I. Porchay-Balderelli, O. Lantieri, B. Balkau, M. Marre and D. S. Group (2011). "Dairy products and the metabolic syndrome in a prospective study, DESIR." J Am Coll Nutr **30**(5 Suppl 1): 454S-463S.

Gami, A. S., B. J. Witt, D. E. Howard, P. J. Erwin, L. A. Gami, V. K. Somers and V. M. Montori (2007). "Metabolic syndrome and risk of incident cardiovascular events and death: a systematic review and meta-analysis of longitudinal studies." J Am Coll Cardiol **49**(4): 403-414.

Grundy, S.M., Cleeman, J.I., Daniels, S.R., Donato, K.A., Eckel, R.H., Franklin, B.A., Gordon, D.J., Krauss, R.M., Savage, P.J., Smith, S.C. Jr, Spertus, J.A. & Costa, F. (2005) “Diagnosis and management of the metabolic syndrome: an American heart association/National Heart, Lung, and Blood Institute Scientific Statement.” Circulation 112, 2735–2752.

Hosseinpour-Niazi, S., P. Mirmiran, Z. Amiri, F. Hosseini-Esfahani, N. Shakeri and F. Azizi (2012). "Legume intake is inversely associated with metabolic syndrome in adults." Arch Iran Med **15**(9): 538-544.

Jafari Nasab, S., Bahrami, A., Rafiee, P., Hekmatdoust, A., Ghanavati, M., Rashidkhani, B., ... &

Hejazi, E. (2019). “Healthy Eating Index-2010 and Mediterranean-Style Dietary Pattern Score and the risk of colorectal cancer and adenoma: a case–control study.” Nutr Cancer, 1-10.

Kang Y, Kim J, “Association between fried food consumption and hypertension in Korean adults.”Br J Nutr. 2016 Jan 14;115(1):87-94

Kang Y, Kim J. (2016), “Gender difference on the association between dietary patterns and metabolic syndrome in Korean population.” Eur J Nutr [Epub ahead of print]

Karageorgou, D., Magriplis, E., Bakogianni, I., Mitsopoulou, A. V., Dimakopoulos, I., Micha, R., & Chourdakis, M. (2019). “Dietary patterns and cardiovascular disease in Greek adults: The Hellenic National Nutrition and Health Survey (HNNHS).” Nutr Metab Cardiovasc Dis

Kim D, Kim J. “Age and sex differences in the relationship between serum 25-hydroxy vitamin D and hypertension in the general Korean population.” Eur J Clin Nutr. 2016 Mar;70(3):326-32.

Kim, J. (2013). “Dairy food consumption is inversely associated with the risk of the metabolic syndrome in Korean adults.” J Hum Nutr Diet 26 Suppl 1: 171-179.

Lutsey PL1, Steffen LM, Stevens J. (2008) “Dietary intake and the development of the metabolic syndrome: the Atherosclerosis Risk in Communities study.” Circulation. 12;117(6):754-61.

McNeill, A. M., W. D. Rosamond, C. J. Girman, S. H. Golden, M. I. Schmidt, H. E. East, C. M. Ballantyne and G. Heiss(2005). “The metabolic syndrome and 11-year risk of incident cardiovascular disease in the atherosclerosis risk in communities study.” Diabetes Care **28**(2): 385-390.

Meigs, J. B., M. K. Rutter, L. M. Sullivan, C. S. Fox, R. B. D'Agostino, Sr. and P. W. Wilson (2007). “Impact of insulin resistance on risk of type 2 diabetes and cardiovascular disease in people with metabolic syndrome.” Diabetes Care **30**(5): 1219-1225.

Milajerdi, A., Namazi, N., Larijani, B., & Azadbakht, L. (2018). “The association of dietary quality indices and cancer mortality: a systematic review and meta-analysis of cohort studies.” Nutr Cancer, 70(7), 1091-1105.

Ministry of Health and Welfare (2005). National Health And Nutrition Examination Survey Report 2005. Seoul, Ministry of Health and Welfare.

Nikniaz, L., Farhangi, M. A., Tabrizi, J. S., & Nikniaz, Z. (2019). “Association of major dietary patterns and different metabolic phenotypes: a population-based study of northwestern Iran.” BMC Endocrine Disorders, 19(1), 131.

Onvani, S., Haghighatdoost, F., Surkan, P. J., Larijani, B., & Azadbakht, L. (2017). “Adherence to the Healthy Eating Index and Alternative Healthy Eating Index dietary patterns and mortality from all causes, cardiovascular disease and cancer: a meta- analysis of observational studies.” J Hum Nutr Diet, 30(2), 216-226.

Satija A, Bhupathiraju SN, Rimm EB, Spiegelman D, Chiuve SE, Borgi L, Willett WC, Manson JE, Sun Q, Hu FB, (2016) “Plant-Based Dietary Patterns and Incidence of Type 2 Diabetes in US

Men and Women: Results from Three Prospective Cohort Studies." PLoS Med. 2016 Jun 14;13(6):e1002039.

Schwingshackl, L., & Hoffmann, G. (2015). "Diet quality as assessed by the Healthy Eating Index, the Alternate Healthy Eating Index, the Dietary Approaches to Stop Hypertension score, and health outcomes: a systematic review and meta-analysis of cohort studies." J Acad Nutr Diet.115(5), 780-800.

Shin, H. J., E. Cho, H. J. Lee, T. T. Fung, E. Rimm, B. Rosner, J. E. Manson, K. Wheelan and F. B. Hu (2014). "Instant noodle intake and dietary patterns are associated with distinct cardiometabolic risk factors in Korea." J Nutr 144(8): 1247-1255.

Shivappa, N., Godos, J., Hébert, J. R., Wirth, M. D., Piuri, G., Speciani, A. F., & Grosso, G. (2017). "Dietary inflammatory index and colorectal cancer risk—A meta-analysis." Nutrients, 9(9), 1043.

Sonestedt E1, Wirfält E, Wallström P, Gullberg B, Orho-Melander M, Hedblad B. (2011). "Dairy products and its association with incidence of cardiovascular disease: the Malmö diet and cancer cohort." Eur J Epidemiol **26**(8):609-618

Song, S., J. E. Lee, W. O. Song, H. Y. Paik and Y. Song (2014). "Carbohydrate intake and refined-grain consumption are associated with metabolic syndrome in the Korean adult population." J Acad Nutr Diet **114**(1): 54-62.

Zaribaf, F., E. Falahi, F. Barak, M. Heidari, A. H. Keshteli, A. Yazdannik and A. Esmaillzadeh (2014). "Fish consumption is inversely associated with the metabolic syndrome." Eur J Clin Nutr **68**(4): 474-480.
