# Supplementary material for: Plant-based diets and incident metabolic syndrome: Results from a South Korean prospective cohort study
Source: PLoS Med. 2020 Nov 18;17(11):e1003371. doi: 10.1371/journal.pmed.1003371 (PMC7673569; doi:10.1371/journal.pmed.1003371)
Supplement: S2 Text — (PDF) [file pmed.1003371.s006.pdf]

국립보건연구원 유전체센터

식품섭취빈도조사

Food Frequency Questionnaire

다음은 지난 1년 동안 드신 음식과 식품에 관한 질문입니다. 각 항목마다 얼마나 자주 드시는지 (지난 1년간 평균 섭취 빈도) 표시하여 주시고 드시는 음식에 대해서만 얼마만큼씩 드시는지(평균 1회 섭취 분량) 해당 칸에 표시해 주시기 바랍니다. 모든 항목에 응답을 하셔야 합니다. 거의 먹지 않는 경우는 섭취분량을 표시하지 않습니다.

The following questions concern foods and beverages consumed in the past year. For each item, please report the usual frequency and amount of consumption (per occassion). You should answer all questions. If you rarely consumed a specific food item,please do not fill in the amount.

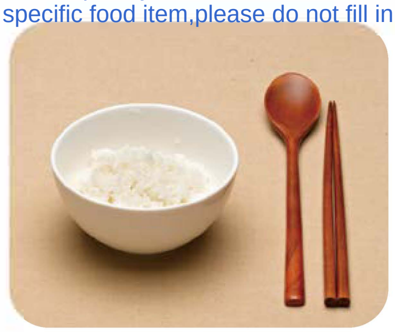

〈 사진 1–1. 밥 〉
Photo 1-1. Rice

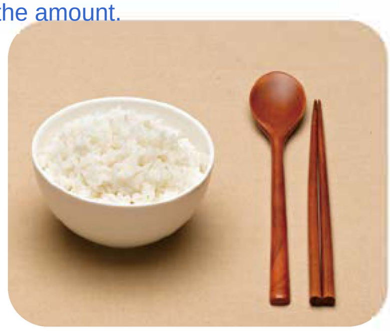

〈 사진 1–2. 밥 〉
Photo 1-2. Rice

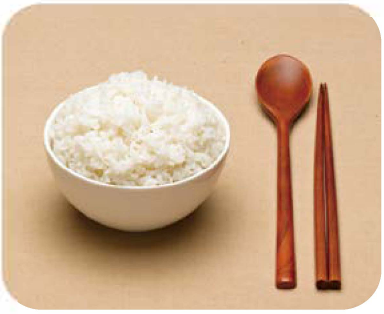

〈 사진 1–3. 밥 〉
Photo 1-3. Rice

Usual consumption frequency in the past year

	지난 1년간 평균 섭취 빈도									Amount consumed per occasion 평균 1회 섭취분량
	거의 안먹음	Monthly 월 1회	월 2~3회	Weekly 주 1~2회	주 3~4회	주 5~6회	Daily 일 1회	일 2회	일 3회	
	Rarely	once	2-3 times	1-2 times	3-4 times	5-6 times	once	twice	3 times	
Grain(Steamed) 밥	☐1	☐2	☐3	☐4	☐5	☐6	☐7	☐8	☐9	☐1 사진 1-1 (1/2공기) Photo 1-1. 1/2 bowl<br>☐2 사진 1-2 (1공기) Photo 1-2. 1 bowl<br>☐3 사진 1-3 (1공기 반) Photo 1-3. 1 1/2 bowl
	주로 드시는 밥의 종류는 ? What type of grain do you usually eat?<br>① 쌀밥 ② 잡곡밥 ③ 쌀밥과 잡곡밥을 비슷하게 먹는다 White rice; mixed grain; similar amount of both<br>잡곡밥의 종류는? What type of mixed grain do you usually eat?<br>① 콩밥 ② 기타잡곡밥 grain with beans; other mixed grain<br>(집에서 잡수시는 것 뿐 아니라 회사나 식당에서 드시는 것도 포함하여 생각하십시오.)<br>Please consider foods eaten in home and foods eaten away from home.									
Instant noodles 라면	☐1	☐2	☐3	☐4	☐5	☐6	☐7	☐8	☐9	☐1 ½그릇 1/2 bowl<br>☐2 1그릇 1 bowl<br>☐3 1그릇 반 1 1/2 bowl
Other noodles (udon noodles) 칼국수/ 장국국수/ 우동	☐1	☐2	☐3	☐4	☐5	☐6	☐7	☐8	☐9	☐1 ½그릇 1/2 bowl<br>☐2 1그릇 1 bowl<br>☐3 1그릇 반 1 1/2 bowl
Black bean sauce noodles 짜장면/ 짬뽕	☐1	☐2	☐3	☐4	☐5	☐6	☐7	☐8	☐9	☐1 ½그릇 1/2 bowl<br>☐2 1그릇 1 bowl<br>☐3 1그릇 반 1 1/2 bowl
Cold noodles 냉면/ 메밀국수	☐1	☐2	☐3	☐4	☐5	☐6	☐7	☐8	☐9	☐1 ½그릇 1/2 bowl<br>☐2 1그릇 1 bowl<br>☐3 1그릇 반 1 1/2 bowl
Dumplings/ dumpling soup 만두/ 만두국	☐1	☐2	☐3	☐4	☐5	☐6	☐7	☐8	☐9	☐1 냉동만두 5개/½그릇 5 dumplings/ 1/2 bowl<br>☐2 냉동만두 10개/1그릇 10 dumplings/ 1 bowl<br>☐3 냉동만두 15개/1그릇 반 15 dumplings/ 1 1/2 bowl
Rice cake/ rice cake soup 흰떡/ 떡국	☐1	☐2	☐3	☐4	☐5	☐6	☐7	☐8	☐9	☐1 ½그릇 1/2 bowl<br>☐2 1그릇 1 bowl<br>☐3 1그릇 반 1 1/2 bowl

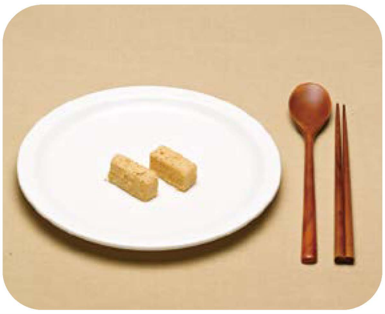

〈 사진 2–1. 떡 〉
Photo 2-1, Rice cake

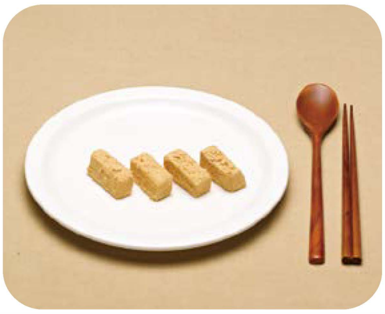

〈 사진 2–2. 떡 〉
Photo 2-2, Rice cake

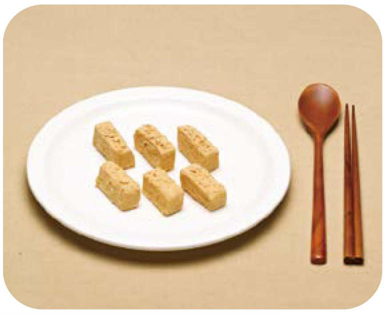

〈 사진 2–3. 떡 〉
Photo 2-3, Rice cake

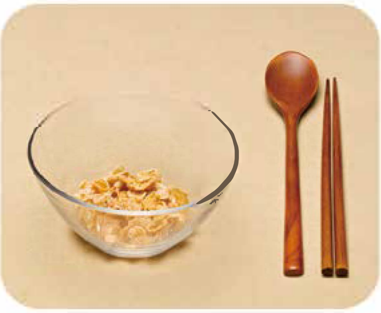

〈 사진 3–1. 콘푸레이크 〉
Photo 3-1, Cereal

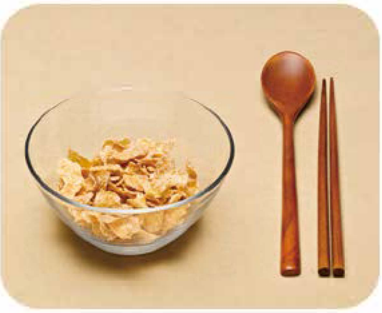

〈 사진 3–2. 콘푸레이크 〉
Photo 3-2, Cereal

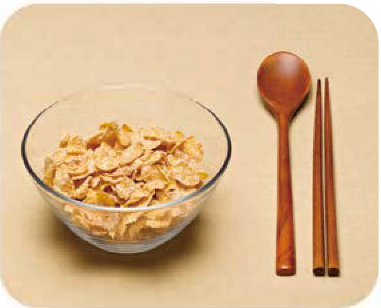

〈 사진 3–3. 콘푸레이크 〉
Photo 3-3, Cereal

	지난 1년간 평균 섭취 빈도 (Usual consumption frequency in the past year)									평균 1회 섭취분량 (Amount consumed per occasion)
	거의 안먹음	월 (Monthly)		주 (Weekly)			일 (Daily)			
		1회	2~3회	1~2회	3~4회	5~6회	1회	2회	3회	
	Rarely	once	2-3 times	1-2 times	3-4 times	5-6 times	once	twice	3 times	
Other rice cakes<br>떡<br>(시루떡, 백설기, 인절미 등)	☐1	☐2	☐3	☐4	☐5	☐6	☐7	☐8	☐9	☐1 사진 2-1 (2개) Photo 2-1, 2 items<br>☐2 사진 2-2(4개) Photo 2-2, 4 items<br>☐3 사진 2-3 (6개) Photo 2-3, 6 items
Cereal<br>콘푸레이크	☐1	☐2	☐3	☐4	☐5	☐6	☐7	☐8	☐9	☐1 사진 3-1 (½그릇) Photo 3-1, 1/2 bowl<br>☐2 사진 3-2 (한그릇) Photo 3-2, 1 bowl<br>☐3 사진 3-3 (한그릇 반) Photo 3-3, 1 1/2 bowl
White breads<br>식빵/ 샌드위치/ 토스트	☐1	☐2	☐3	☐4	☐5	☐6	☐7	☐8	☐9	☐1 1쪽 1 slice<br>☐2 2쪽 2 slices<br>☐3 3쪽 3 slices
Butter/margarine<br>잼/ 꿀/ 버터/ 마아가린<br>(빵에 발라먹는 경우)	☐1	☐2	☐3	☐4	☐5	☐6	☐7	☐8	☐9	☐1 ½찻술 1/2 teaspoon<br>☐2 1찻술 1 teaspoon<br>☐3 2찻술 2 teaspoon
Sweet red bean bread<br>팥빵/ 호빵/ 풀빵	☐1	☐2	☐3	☐4	☐5	☐6	☐7	☐8	☐9	☐1 ½개 1/2 item<br>☐2 1개 1 item<br>☐3 1개 반 1 1/2 items
Other breads<br>기타 빵<br>(곰보빵, 도우넛,<br>카스테라, 크림빵)	☐1	☐2	☐3	☐4	☐5	☐6	☐7	☐8	☐9	☐1 ½개 1/2 item<br>☐2 1개 1 item<br>☐3 1개 반 1 1/2 itmes

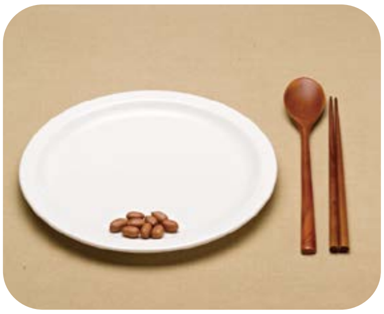

〈 사진 4–1. 땅콩 〉
Photo 4-1, Peanuts

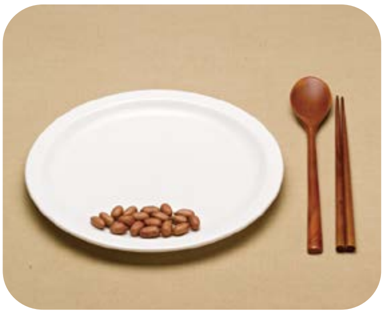

〈 사진 4–2. 땅콩 〉
Photo 4-2, Peanuts

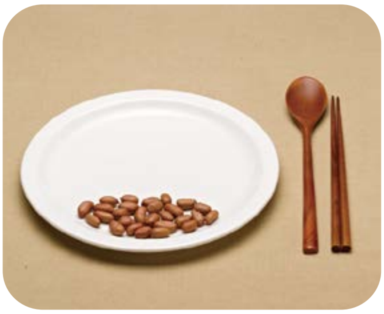

〈 사진 4–3. 땅콩 〉
Photo 4-3, Peanuts

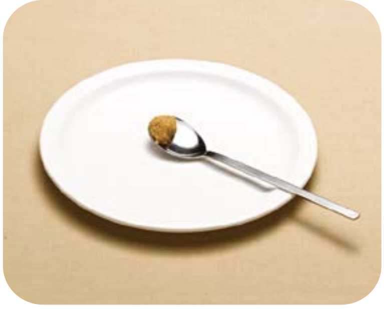

〈 사진 5–1. 된장 〉
Photo 5-1, Bean paste

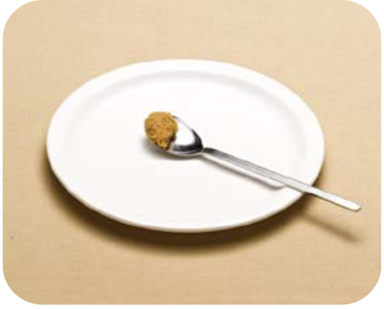

〈 사진 5–2. 된장 〉
Photo 5-2, Bean paste

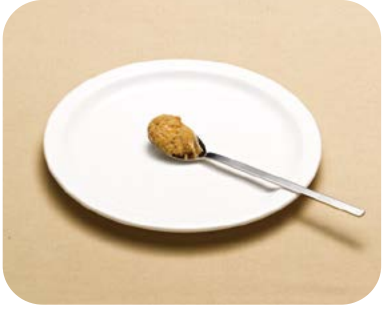

〈 사진 5–3. 된장 〉
Photo 5-3, Bean paste

	지난 1년간 평균 섭취 빈도									평균 1회 섭취분량
	거의 안먹음	월		주			일			
		1회	2~3회	1~2회	3~4회	5~6회	1회	2회	3회	
Cake / chocolated pie<br>케익/초코파이	☐1	☐2	☐3	☐4	☐5	☐6	☐7	☐8	☐9	☐1 ½개 1/2 item<br>☐2 1개 1 item<br>☐3 1개 반 1 1/2 items
Pizza / Hamburger<br>피자/햄버거	☐1	☐2	☐3	☐4	☐5	☐6	☐7	☐8	☐9	☐1 큰 것 ½개 1/2 item<br>☐2 큰 것 1개 1 item<br>☐3 큰 것 1개 반 1 1/2 items
Grain powder<br>선식/미숫가루	☐1	☐2	☐3	☐4	☐5	☐6	☐7	☐8	☐9	☐1 2큰술 2 tablespoon<br>☐2 3큰술/선식 1봉지 3 tablespoon/ grain powder 1 bag<br>☐3 4큰술 4 tablespoon
Cookies / Crakers / Snacks<br>쿠키/ 크래커/ 스넥	☐1	☐2	☐3	☐4	☐5	☐6	☐7	☐8	☐9	☐1 반봉지 이하 less than half bag<br>☐2 반봉지 1/2 bag<br>☐3 1봉지 1 bag
Candies / Chocolates<br>사탕/ 초콜릿	☐1	☐2	☐3	☐4	☐5	☐6	☐7	☐8	☐9	☐1 3개/반개 3 items/ 1/2 item<br>☐2 5개/한개 5 items/ 1 item<br>☐3 7개/한개 반 7 items/ 1 1/2 items
Peanuts / Almonds / Pine nuts<br>땅콩/ 아몬드/ 잣	☐1	☐2	☐3	☐4	☐5	☐6	☐7	☐8	☐9	☐1 사진 4-1 (반줌) Photo 4-1, 1/2 handful<br>☐2 사진 4-2 (한줌) Photo 4-2, 1 handful<br>☐3 사진 4-3 (한줌 반) Photo 4-3, 1 1/2 handful
Beans / Beans cooked in soy sauce<br>콩/ 콩자반<br>(초콩포함, 콩밥제외)	☐1	☐2	☐3	☐4	☐5	☐6	☐7	☐8	☐9	☐1 사진 4-1 (½줌) Photo 4-1, 1/2 handful<br>☐2 사진 4-2 (한줌) Photo 4-2, 1 handful<br>☐3 사진 4-3 (한줌 반) Photo 4-3, 1 1/2 handful
Bean paste / Bean paste soup<br>된장국/ 청국장/<br>된장/ 쌈장	☐1	☐2	☐3	☐4	☐5	☐6	☐7	☐8	☐9	☐1 사진 5-1 (½그릇) Photo 5-1, 1/2 bowl<br>☐2 사진 5-2 (1그릇) Photo 5-2, 1 bowl<br>☐3 사진 5-3 (1그릇 반) Photo 5-3, 1 1/2 bowl

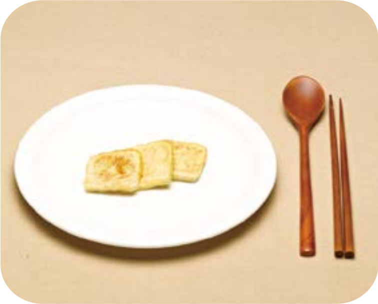

〈 사진 6–1. 두부 〉
Photo 6-1, Tofu

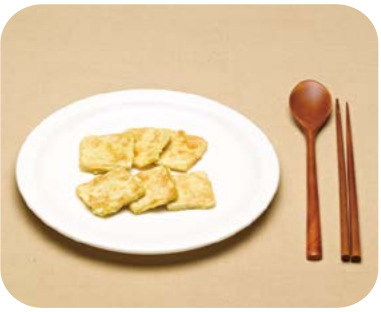

〈 사진 6–2. 두부 〉
Photo 6-2, Tofu

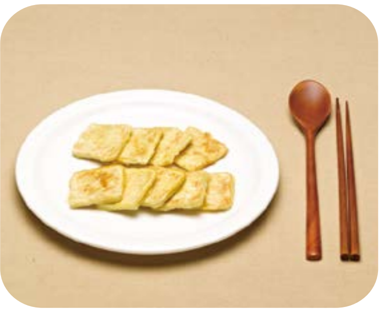

〈 사진 6–3. 두부 〉
Photo 6-3, Tofu

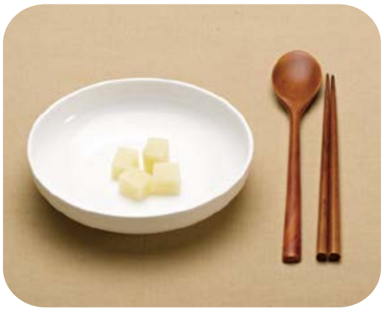

〈 사진 7–1. 감자 〉
Photo 7-1, Potatoes

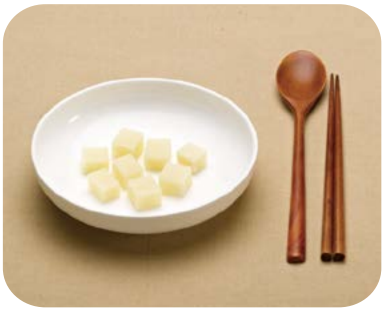

〈 사진 7–3. 감자 〉
Photo 7-2, Potatoes

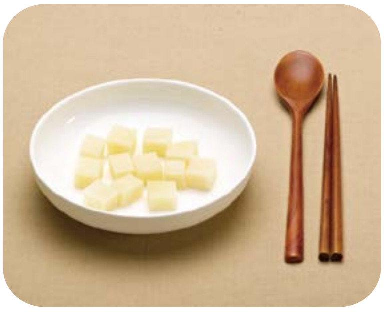

〈 사진 7–3. 감자 〉
Photo 7-3, Potatoes

	지난 1년간 평균 섭취 빈도									평균 1회 섭취분량
	거의 안먹음	월		주			일			
		1회	2~3회	1~2회	3~4회	5~6회	1회	2회	3회	
Eggs / Quail eggs<br>계란/ 메추리알	☐1	☐2	☐3	☐4	☐5	☐6	☐7	☐8	☐9	☐1 계란 ½개/메추리알 3개 1/2 egg/ 3 quail eggs<br>☐2 계란 1개/메추리알 5개 1 egg/ 5 quail eggs<br>☐3 계란 1개 반/메추리알 8개 1 1/2 eggs/ 8 quail eggs
두 부 Tofu<br>(순두부, 두부찌개, 된장찌개에 들어간 두부 포함)	☐1	☐2	☐3	☐4	☐5	☐6	☐7	☐8	☐9	☐1 사진 6-1 (3쪽) Photo 6-1, 3 slices<br>☐2 사진 6-2 (6쪽) Photo 6-2, 6 slices<br>☐3 사진 6-3 (9쪽) Photo 6-3, 9 slices
Starch Jelly<br>묵	☐1	☐2	☐3	☐4	☐5	☐6	☐7	☐8	☐9	☐1 사진 6-1 (3쪽) Photo 6-1, 3slices<br>☐2 사진 6-2 (6쪽) Photo 6-2, 6 slices<br>☐3 사진 6-3 (9쪽) Photo 6-3, 9 slices
감 자 Potatoes<br>(찐감자, 감자튀김, 감자국, 감자찌개, 감자지짐 등)	☐1	☐2	☐3	☐4	☐5	☐6	☐7	☐8	☐9	☐1 사진 7-1 (중 ⅛개) Photo 7-1, medium size 1/8 item<br>☐2 사진 7-2 (중 ¼개) Photo 7-2, medium size 1/4 item<br>☐3 사진 7-3 (중 ⅓개) Photo 7-3 medium size 1/3 item
Sweet potatoes<br>고구마<br>(찐고구마, 맛탕 등)	☐1	☐2	☐3	☐4	☐5	☐6	☐7	☐8	☐9	☐1 중 ½개 Medium size 1/2 item<br>☐2 중 1개 Medium size 1 item<br>☐3 중 1개 반 Medium size 1 1/2 items
Stir-fried noodles and vegetables<br>잡채 (당면)	☐1	☐2	☐3	☐4	☐5	☐6	☐7	☐8	☐9	☐1 사진 10-1 (½접시) Photo 10-1, 1/2 plate<br>☐2 사진 10-2 (1접시) Photo 10-2, 1 plate<br>☐3 사진 10-3 (1접시 반) Photo 10-3 1 1/2 plates

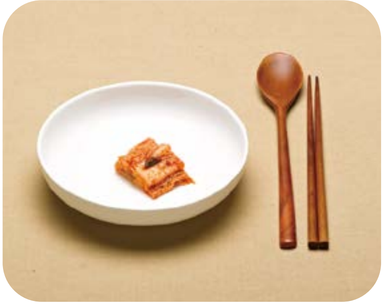

〈 사진 8–1. 김치 〉
Photo 8-1, Kimchi

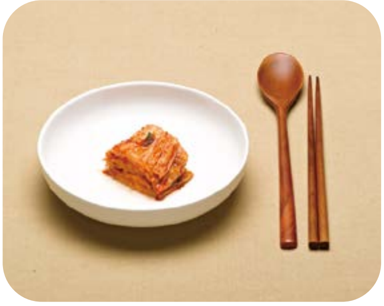

〈 사진 8–2. 김치 〉
Photo 8-2, Kimchi

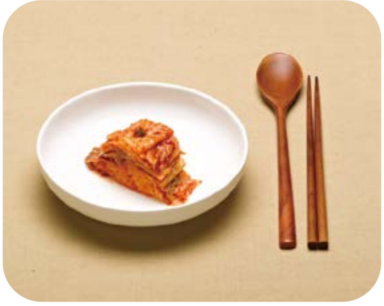

〈 사진 8–3. 김치 〉
Photo 8-3, Kimchi

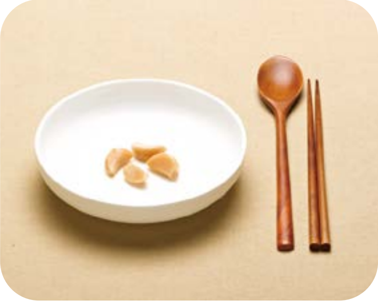

〈 사진 9–1. 장아찌 〉
Photo 9-1, Pickled vegetable

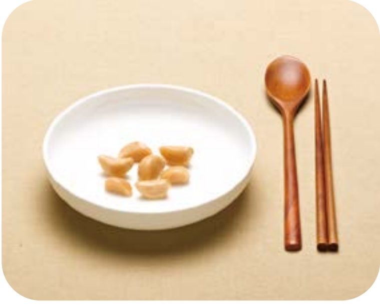

〈 사진 9–2. 장아찌 〉
Photo 9-2, Pickled vegetable

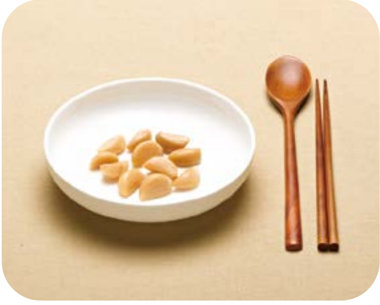

〈 사진 9–3. 장아찌 〉
Photo 9-3, Pickled vegetable

	지난 1년간 평균 섭취 빈도									평균 1회 섭취분량
	거의 안먹음	월		주			일			
Korean cabbage kimchi		1회	2~3회	1~2회	3~4회	5~6회	1회	2회	3회	
배추김치<br>(배추김치, 백김치,<br>김치찌개 속 배추김치 등)	□1	□2	□3	□4	□5	□6	□7	□8	□9	□1 사진 8-1 (3쪽) Photo 8-1, 3 slices<br>□2 사진 8-2 (6쪽) Photo 8-2, 6 slices<br>□3 사진 8-3 (9쪽) Photo 8-3, 9 slices
Radish kimchi<br>깍두기/ 무김치	□1	□2	□3	□4	□5	□6	□7	□8	□9	□1 사진 8-1 (3개) Photo 8-1, 3 items<br>□2 사진 8-2 (5개) Photo 8-2, 5 items<br>□3 사진 8-3 (8개) Photo 8-3, 8 items
Radish<br>무(국,조림)/ 단무지	□1	□2	□3	□4	□5	□6	□7	□8	□9	□1 사진 8-1 Photo 8-1<br>□2 사진 8-2 Photo 8-2<br>□3 사진 8-3 Photo 8-3
Other Kimchi<br>기타김치<br>(고들빼기, 갓김치, 파김치)	□1	□2	□3	□4	□5	□6	□7	□8	□9	□1 사진 8-1 Photo 8-1<br>□2 사진 8-2 Photo 8-2<br>□3 사진 8-3 Photo 8-3
Pickled vegetable<br>장아찌<br>(마늘장아찌, 마늘쫑, 무장아찌)	□1	□2	□3	□4	□5	□6	□7	□8	□9	□1 사진 9-1 Photo 9-1<br>□2 사진 9-2 Photo 9-2<br>□3 사진 9-3 Photo 9-3
Watery radish kimchi<br>나박김치/ 동치미	□1	□2	□3	□4	□5	□6	□7	□8	□9	□1 ½대접 1/2 bowl<br>□2 1대접 1 bowl<br>□3 1대접 반 1 1/2 bowl
Napa cabbage / Napa cabbage soup (Korean cabbage / Korean cabbage soup)<br>배추/ 배추국	□1	□2	□3	□4	□5	□6	□7	□8	□9	□1 ½그릇 (½장) 1/2 bowl (1/2 piece)<br>□2 1그릇 (1장) 1 bowl (1 piece)<br>□3 1그릇 반 (1장 반) 1 1/2 bowl (1 1/2 pieces)

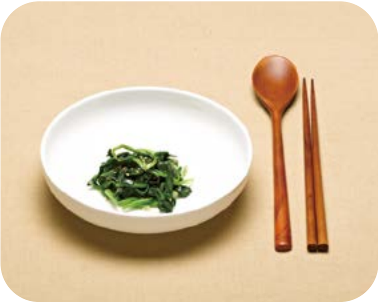

〈 사진 10–1. 나물무침 〉
Photo 10-1, Seasoned vegetables

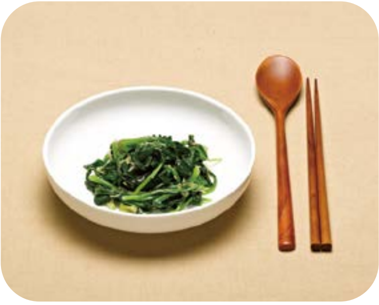

〈 사진 10–2. 나물무침 〉
Photo 10-2, Seasoned vegetables

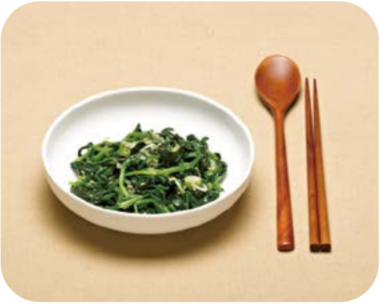

〈 사진 10–3. 나물무침 〉
Photo 10-3, Seasoned vegetables

	지난 1년간 평균 섭취 빈도									평균 1회 섭취분량
	거의 안먹음	월		주			일			
		1회	2~3회	1~2회	3~4회	5~6회	1회	2회	3회	
시금치 Spinach<br>(시금치 나물, 국 등)	□1	□2	□3	□4	□5	□6	□7	□8	□9	□1 사진 10-1 Photo 10-1<br>□2 사진 10-2 Photo 10-2<br>□3 사진 10-3 Photo 10-3
상 추 Lettus<br>(쌈, 무침 등)	□1	□2	□3	□4	□5	□6	□7	□8	□9	□1 손바닥크기 2장 2 pieces<br>□2 손바닥크기 4장 4 pieces<br>□3 손바닥크기 6장 6 pieces
Perilla leaves<br>들깻잎	□1	□2	□3	□4	□5	□6	□7	□8	□9	□1 3장 3 pieces<br>□2 6장 6 pieces<br>□3 9장 9 pieces
야채쌈/ 야채샐러드 Sesame leaves / Vegetables salad<br>(양배추,양상치,케일,치커리,<br>청경채,브로콜리 등)	□1	□2	□3	□4	□5	□6	□7	□8	□9	□1 큰 것 1장 1 piece<br>□2 큰 것 2장 2 pieces<br>□3 큰 것 3장 3 pieces
Other green vegetable<br>기타 녹색채소<br>(냉이, 근대, 아욱, 쑥, 우거지 등)	□1	□2	□3	□4	□5	□6	□7	□8	□9	□1 사진 10-1 Photo 10-1<br>□2 사진 10-2 Photo 10-2<br>□3 사진 10-3 Photo 10-3
Deodeok / Bellflower root<br>더덕/도라지	□1	□2	□3	□4	□5	□6	□7	□8	□9	□1 사진 10-1 Photo 10-1<br>□2 사진 10-2 Photo 10-2<br>□3 사진 10-3 Photo 10-3
Bean sprouts / Mung-bean sprouts<br>콩나물/숙주나물	□1	□2	□3	□4	□5	□6	□7	□8	□9	□1 사진 10-1 Photo 10-1<br>□2 사진 10-2 Photo 10-2<br>□3 사진 10-3 Photo 10-3
Bracken / Sweet potato stem / Taro stem<br>고사리/ 고구마줄기/ 토란대	□1	□2	□3	□4	□5	□6	□7	□8	□9	□1 사진 10-1 Photo 10-1<br>□2 사진 10-2 Photo 10-2<br>□3 사진 10-3 Photo 10-3
Oyster Mushrooms<br>느타리버섯	□1	□2	□3	□4	□5	□6	□7	□8	□9	□1 사진 10-1 Photo 10-1<br>□2 사진 10-2 Photo 10-2<br>□3 사진 10-3 Photo 10-3
Other mushrooms<br>기타버섯<br>(목이, 양송이, 팽이 등)	□1	□2	□3	□4	□5	□6	□7	□8	□9	□1 사진 10-1 Photo 10-1<br>□2 사진 10-2 Photo 10-2<br>□3 사진 10-3 Photo 10-3

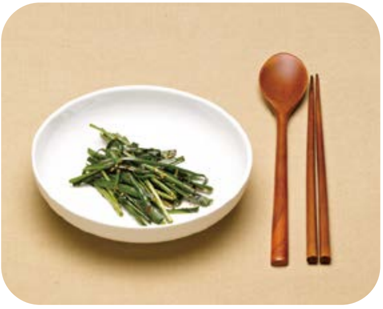

〈 사진 11–1. 부추 〉
Photo 11-1, Chives

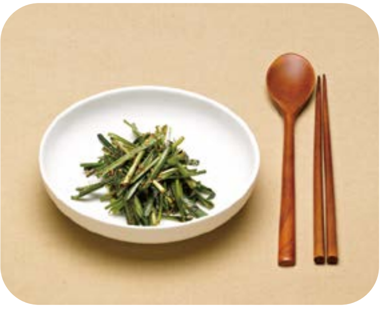

〈 사진 11–2. 부추 〉
Photo 11-2, Chives

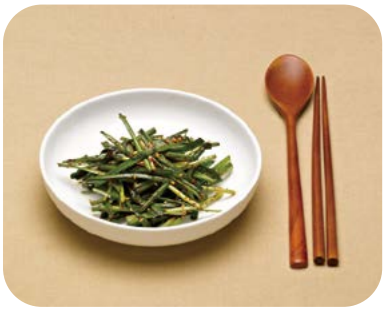

〈 사진 11–3. 부추 〉
Photo 11-3, Chives

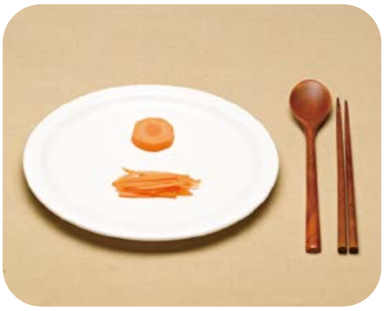

〈 사진 12–1. 당근 〉
Photo 12-1, Carrot

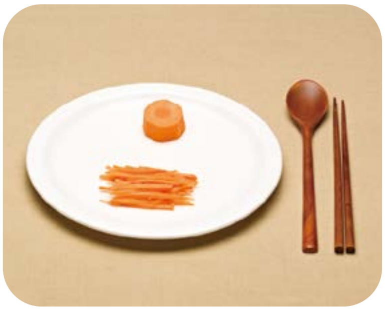

〈 사진 12–2. 당근 〉
Photo 12-2 Carrot

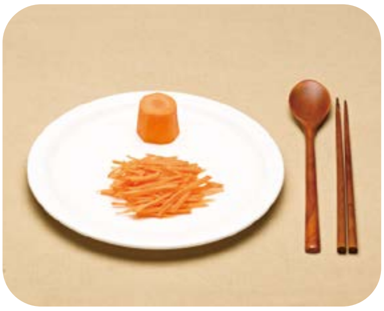

〈 사진 12–3. 당근 〉
Photo 12-3 Carrot

	지난 1년간 평균 섭취 빈도									평균 1회 섭취분량
	거의 안먹음	월		주			일			
		1회	2~3회	1~2회	3~4회	5~6회	1회	2회	3회	
Green pepper leaf / Chamnamul / Wild aster (Chwinamul)<br>고춧잎/ 참나물/ 취나물	□1	□2	□3	□4	□5	□6	□7	□8	□9	□1 사진 11-1 Photo 11-1<br>□2 사진 11-2 Photo 11-2<br>□3 사진 11-3 Photo 11-3
Crown daisy / Chives / Water cress<br>쑥갓/ 부추/ 미나리	□1	□2	□3	□4	□5	□6	□7	□8	□9	□1 사진 11-1 Photo 11-1<br>□2 사진 11-2 Photo 11-2<br>□3 사진 11-3 Photo 11-3
Cucumber<br>오이	□1	□2	□3	□4	□5	□6	□7	□8	□9	□1 사진 12-1 Photo 12-1<br>□2 사진 12-2 Photo 12-2<br>□3 사진 12-3 Photo 12-3
Carrot / Carrot juice<br>당근/ 당근주스	□1	□2	□3	□4	□5	□6	□7	□8	□9	□1 사진 12-1 Photo 12-1<br>□2 사진 12-2 Photo 12-2<br>□3 사진 12-3 Photo 12-3
Onion<br>양파	□1	□2	□3	□4	□5	□6	□7	□8	□9	□1 사진 12-1 Photo 12-1<br>□2 사진 12-2 Photo 12-2<br>□3 사진 12-3 Photo 12-3
Green peppers<br>풋고추	□1	□2	□3	□4	□5	□6	□7	□8	□9	□1 1개 1 item<br>□2 2개 2 items<br>□3 3개 3 items
Zucchini<br>애호박	□1	□2	□3	□4	□5	□6	□7	□8	□9	□1 사진 12-1 Photo 12-1<br>□2 사진 12-2 Photo 12-2<br>□3 사진 12-3 Photo 12-3
Pumpkin / Kabocha squash<br>늙은호박/ 단호박/ 호박즙	□1	□2	□3	□4	□5	□6	□7	□8	□9	□1 죽 ½그릇/ ½팩 1/2 bowl/1/2 pack<br>□2 죽 한그릇/1팩 1 bowl/1pack<br>□3 죽 한그릇 반/1팩 반 1 1/2 bowl/ 1 1/2 packs

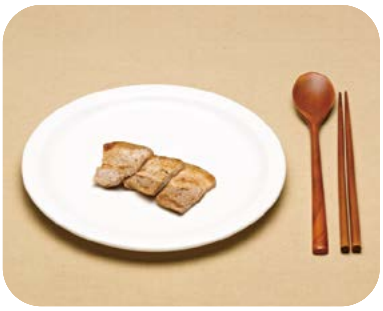

〈 사진 13–1. 고기 75그램 〉
Photo 13-1, Pork belly 75 grams

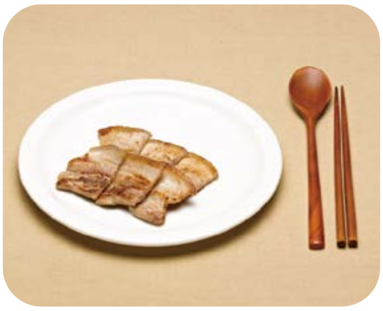

〈 사진 13–2. 고기 150그램 〉
Photo 13-2, Pork belly 150 grams

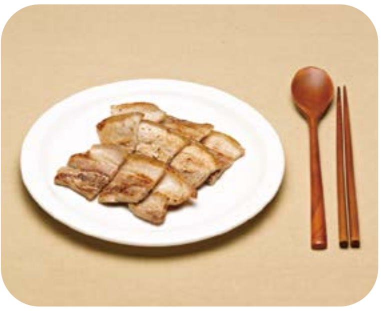

〈 사진 13–3. 고기 225그램 〉
Photo 13-3, Pork belly 225 grams

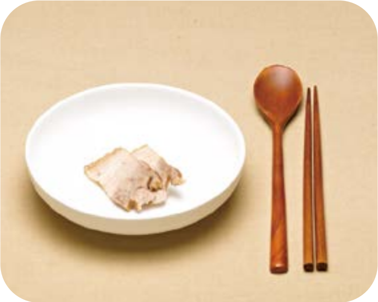

〈 사진 14–1. 돼지고기찜 25그램 〉
Photo 14-1, Steamed pork 25 grams

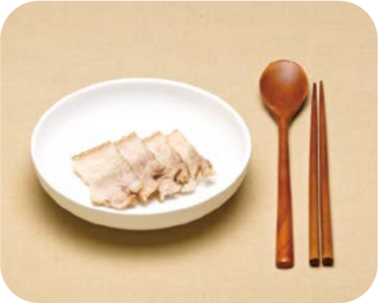

〈 사진 14–2. 돼지고기찜 50그램 〉
Photo 14-2, Steamed pork 50 grams

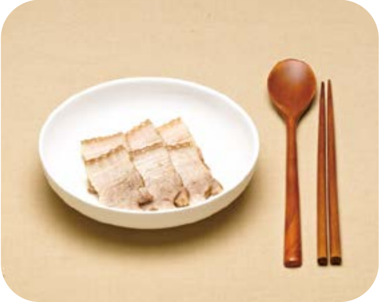

〈 사진 14–3. 돼지고기찜 75그램 〉
Photo 14-3, Steamed pork 75 grams

	지난 1년간 평균 섭취 빈도									평균 1회 섭취분량
	거의 안먹음	월		주			일			
		1회	2~3회	1~2회	3~4회	5~6회	1회	2회	3회	
Pork belly<br>삼겹살	☐1	☐2	☐3	☐4	☐5	☐6	☐7	☐8	☐9	☐1 사진 13-1 Photo 13-1<br>☐2 사진 13-2 Photo 13-2<br>☐3 사진 13-3 Photo 13-3
Grilled pork / Stir-fried Pork / Pork Bulgogi / Korean meatball<br>돼지고기구이/ 볶음/<br>돼지불고기/ 동그랑땡	☐1	☐2	☐3	☐4	☐5	☐6	☐7	☐8	☐9	☐1 사진 13-1 Photo 13-1<br>☐2 사진 13-2 Photo 13-2<br>☐3 사진 13-3 Photo 13-3
스테이크/ 쇠고기구이 Steak / Grilled beef<br>(갈비, 등심, 안심,<br>쇠고기불고기)	☐1	☐2	☐3	☐4	☐5	☐6	☐7	☐8	☐9	☐1 사진 13-1 Photo 13-1<br>☐2 사진 13-2 Photo 13-2<br>☐3 사진 13-3 Photo 13-3
Dog meat<br>개고기	☐1	☐2	☐3	☐4	☐5	☐6	☐7	☐8	☐9	☐1 사진 13-1 Photo 13-1<br>☐2 사진 13-2 Photo 13-2<br>☐3 사진 13-3 Photo 13-3
Steamed pork<br>돼지고기 찜<br>(보쌈, 장조림, 족발)	☐1	☐2	☐3	☐4	☐5	☐6	☐7	☐8	☐9	☐1 사진 14-1 Photo 14-1<br>☐2 사진 14-2 Photo 14-2<br>☐3 사진 14-3 Photo 14-3
Processed meat(ham, sausage)<br>가공육<br>(햄, 소시지)	☐1	☐2	☐3	☐4	☐5	☐6	☐7	☐8	☐9	☐1 비엔나햄 3개, 햄 ½장 3 sausages/1/2 piece ham<br>☐2 비엔나햄 5개, 햄 1장 5 sausages/1 piece ham<br>☐3 비엔나햄 8개, 햄 1장 반 8 sausages/1 1/2 pieces ham
Organ meats/Korean sausage<br>부산물<br>(내장육, 선지, 순대)	☐1	☐2	☐3	☐4	☐5	☐6	☐7	☐8	☐9	☐1 사진 14-1 Photo 14-1<br>☐2 사진 14-2 Photo 14-2<br>☐3 사진 14-3 Photo 14-3

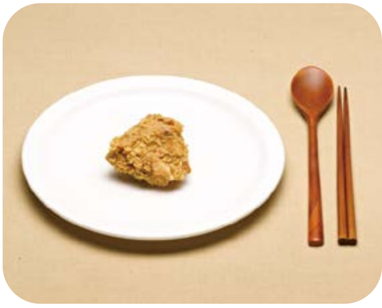

〈 사진 15–1. 닭고기 37.5그램 〉
Photo 15-1, Chicken 37.5 grams

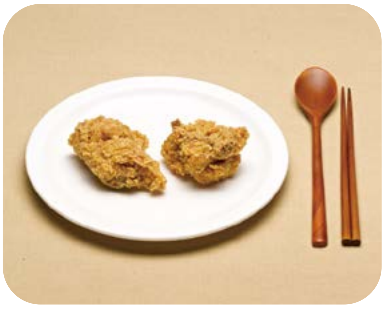

〈 사진 15–1. 닭고기 75그램 〉
Photo 15-1, Chicken 75 grams

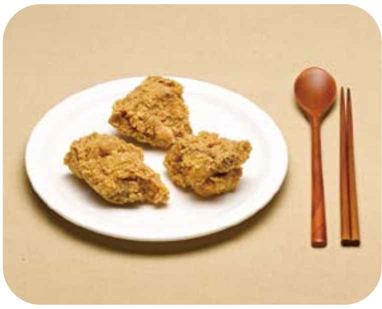

〈 사진 15–1. 닭고기 112.5그램 〉
Photo 15-1, Chicken 112.5 grams

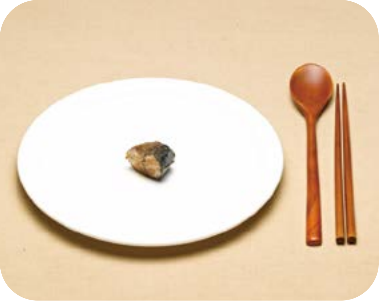

〈 사진 16–1. 생선류 〉
Photo 16-1, Fish

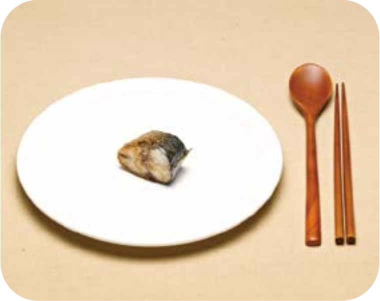

〈 사진 16–2. 생선류 〉
Photo 16-2, Fish

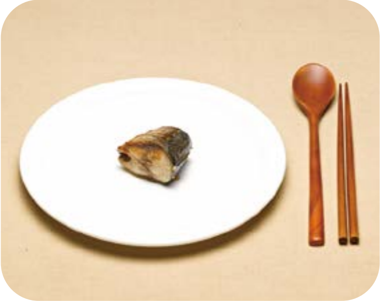

〈 사진 16–3. 생선류 〉
Photo 16-3, Fish

	지난 1년간 평균 섭취 빈도									평균 1회 섭취분량
	거의 안먹음	월		주			일			
		1회	2~3회	1~2회	3~4회	5~6회	1회	2회	3회	
Fried chicken / Chicken stew / Braised Spicy Chicken<br>프라이드치킨/ 닭백숙/ 삼계탕/ 닭도리탕	☐1	☐2	☐3	☐4	☐5	☐6	☐7	☐8	☐9	☐1 사진 15-1 Photo 15-1<br>☐2 사진 15-2 Photo 15-2<br>☐3 사진 15-3 Photo 15-3
탕류 Beef stew<br>(설렁탕, 곰탕, 갈비탕, 도가니탕 등)	☐1	☐2	☐3	☐4	☐5	☐6	☐7	☐8	☐9	☐1 반그릇 1/2 bowl<br>☐2 한그릇 1 bowl<br>☐3 한그릇 반 1 1/2 bowl
국류 Beef soup<br>(쇠고기국, 육개장 등)	☐1	☐2	☐3	☐4	☐5	☐6	☐7	☐8	☐9	☐1 반그릇 1/2 bowl<br>☐2 한그릇 1 bowl<br>☐3 한그릇 반 1 1/2 bowl
Sashimi<br>생선회	☐1	☐2	☐3	☐4	☐5	☐6	☐7	☐8	☐9	☐1 사진 16-1 Photo 16-1<br>☐2 사진 16-2 Photo 16-2<br>☐3 사진 16-3 Photo 16-3
mackerel/pacific saury<br>등푸른생선 (고등어,꽁치,삼치 등)	☐1	☐2	☐3	☐4	☐5	☐6	☐7	☐8	☐9	☐1 사진 16-1 Photo 16-1<br>☐2 사진 16-2 Photo 16-2<br>☐3 사진 16-3 Photo 16-3
Belt fish<br>갈치	☐1	☐2	☐3	☐4	☐5	☐6	☐7	☐8	☐9	☐1 사진 16-1 Photo 16-1<br>☐2 사진 16-2 Photo 16-2<br>☐3 사진 16-3 Photo 16-3
Eel<br>장어	☐1	☐2	☐3	☐4	☐5	☐6	☐7	☐8	☐9	☐1 사진 16-1 Photo 16-1<br>☐2 사진 16-2 Photo 16-2<br>☐3 사진 16-3 Photo 16-3

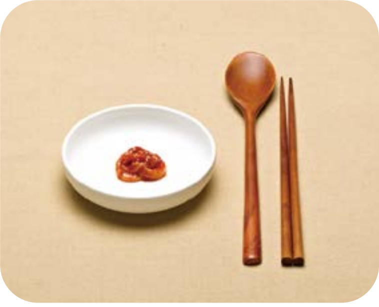

〈 사진 17–1. 젓갈 〉
Photo 17-1, Salted fish

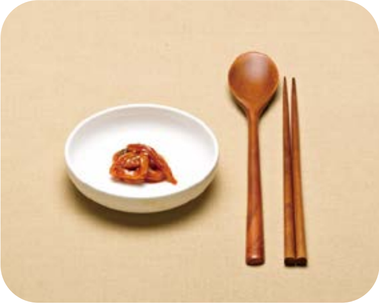

〈 사진 17–2. 젓갈 〉
Photo 17-2, Salted fish

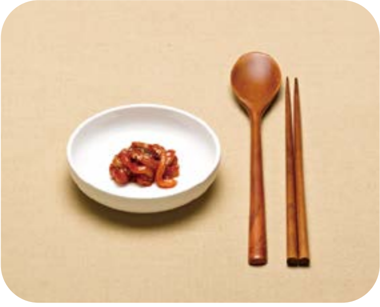

〈 사진 17–3. 젓갈 〉
Photo 17-3, Salted fish

	지난 1년간 평균 섭취 빈도									평균 1회 섭취분량
	거의 안먹음	월		주			일			
		1회	2~3회	1~2회	3~4회	5~6회	1회	2회	3회	
Yellow croaker / Sea Bream / Sole<br>조기/ 돔/ 가자미	☐1	☐2	☐3	☐4	☐5	☐6	☐7	☐8	☐9	☐1 사진 16-1 Photo 16-1<br>☐2 사진 16-2 Photo 16-2<br>☐3 사진 16-3 Photo 16-3
Alaska pollack / Frozen pollack / Dried pollack<br>명태/ 동태/ 북어	☐1	☐2	☐3	☐4	☐5	☐6	☐7	☐8	☐9	☐1 사진 16-1 Photo 16-1<br>☐2 사진 16-2 Photo 16-2<br>☐3 사진 16-3 Photo 16-3
Squid / Dried squid / Octopus<br>오징어/ 마른오징어/ 낙지	☐1	☐2	☐3	☐4	☐5	☐6	☐7	☐8	☐9	☐1 사진 16-1 Photo 16-1<br>☐2 사진 16-2 Photo 16-2<br>☐3 사진 16-3 Photo 16-3
Anchovy / Stir-fried anchovy<br>멸치/ 멸치볶음	☐1	☐2	☐3	☐4	☐5	☐6	☐7	☐8	☐9	☐1 사진 9-1(½큰술) Photo 9-1, 1/2 tablespoon<br>☐2 사진 9-2(1큰술) Photo 9-2, 1 tablespoon<br>☐3 사진 9-3(1과½큰술) Photo 9-3, 1 1/2 tablespoon
Canned tunna<br>참치(캔)	☐1	☐2	☐3	☐4	☐5	☐6	☐7	☐8	☐9	☐1 작은캔 ½개 1/2 small can<br>☐2 작은캔 1개 1 small can<br>☐3 작은캔 1개 반 1 1/2 small cans
젓갈 Salted shrimp/salted fish<br>(오징어젓, 창란젓, 명란젓,<br>새우젓, 멸치젓, 조개젓 등)	☐1	☐2	☐3	☐4	☐5	☐6	☐7	☐8	☐9	☐1 사진 17-1 Photo 17-1<br>☐2 사진 17-2 Photo 17-2<br>☐3 사진 17-3 Photo 17-3
조개/골뱅이 Clam / Sea snail<br>(국, 찌개, 구이, 칼국수,<br>무침 등 포함)	☐1	☐2	☐3	☐4	☐5	☐6	☐7	☐8	☐9	☐1 사진 17-1 Photo 17-1<br>☐2 사진 17-2 Photo 17-2<br>☐3 사진 17-3 Photo 17-3
굴 Oyster<br>(어리굴젓 포함)	☐1	☐2	☐3	☐4	☐5	☐6	☐7	☐8	☐9	☐1 2개 2 items<br>☐2 4개 4 items<br>☐3 6개 6 items
Crab / Marinated Crab<br>게/ 게장	☐1	☐2	☐3	☐4	☐5	☐6	☐7	☐8	☐9	☐1 중 ¼마리 medium size 1/4 item<br>☐2 중 ½마리 medium size 1/2 item<br>☐3 중 1마리 medium size 1 item
shrimp<br>새우	☐1	☐2	☐3	☐4	☐5	☐6	☐7	☐8	☐9	☐1 중 ½마리 medium size 1/2 item<br>☐2 중 1마리 medium size 1 item<br>☐3 중 2마리 medium size 2 items

국립보건연구원 유전체센터

Usual consumption frequency in the past year

	지난 1년간 평균 섭취 빈도									Amount consumed per occasion 평균 1회 섭취분량
		Monthly 월		Weekly 주			Daily 일			
	거의 안먹음 Rarely	1회 once	2~3회 2 3 times	1~2회 1-2 times	3~4회 3-4 times	5~6회 5-6 times	1회 once	2회 twice	3회 3 times	
Fish cake 어묵/게맛살	□1	□2	□3	□4	□5	□6	□7	□8	□9	□1 ⅓개 1/3 item<br>□2 ⅔개 2/3 item<br>□3 1개 1 item
Laver 김	□1	□2	□3	□4	□5	□6	□7	□8	□9	□1 큰것 ½장 (8절 4장) large size 1/2 piece<br>□2 큰것 한 장 (8절 8장) large size 1 piece<br>□3 큰것 한장 반 (8절 12장) large size 1 1/2 pieces
Kelp / Seaweed 다시마/ 미역	□1	□2	□3	□4	□5	□6	□7	□8	□9	□1 국 ½그릇 1/2 bowl<br>□2 국 1 그릇 1 bowl<br>□3 국 한그릇 반 1 1/2 bowl
Milk 우유	□1	□2	□3	□4	□5	□6	□7	□8	□9	□1 ½컵 (100ml) 1/2 cup (100ml)<br>□2 1컵 (200ml) 1 cup (200ml)<br>□3 2컵 (400ml) 2 cups (400ml)
Yogurt / Yoplait 요구르트/ 요플레	□1	□2	□3	□4	□5	□6	□7	□8	□9	□1 ½개 1/2 item<br>□2 1개 1 item<br>□3 2개 2 items
Ice cream 아이스크림	□1	□2	□3	□4	□5	□6	□7	□8	□9	□1 ½개 1/2 item<br>□2 1개 1 item<br>□3 2개 2 items
Cheese 치즈	□1	□2	□3	□4	□5	□6	□7	□8	□9	□1 ½장 1/2 slice<br>□2 1장 1 slice<br>□3 2장 2 slices
Soybean milk 두유	□1	□2	□3	□4	□5	□6	□7	□8	□9	□1 ½컵 (100ml) 1/2 cup (100ml)<br>□2 1컵 (200ml) 1 cup (200ml)<br>□3 2컵 (400ml) 2 cups (400ml)

Usual consumption frequency in the past year

	지난 1년간 평균 섭취 빈도									Amount consumed per occasion 평균 1회 섭취분량
		Monthly 월		Weekly 주			Daily 일			
	거의 안먹음 Rarely	1회 once	2~3회 2-3 times	1~2회 1-2 times	3~4회 3-4 times	5~6회 5-6 times	1~2회 1-2 times	3~4회 3-4 times	5~6회 5-6 times	
Coffe 커피	□1	□2	□3	□4	□5	□6	□7	□8	□9	□1 ½잔 1/2 cup<br>□2 1잔 1 cup<br>□3 2잔 2 cups
Added sugar to tea or coffee 차에 넣는 설탕	□1	□2	□3	□4	□5	□6	□7	□8	□9	□1 1찻술 1 teaspoon<br>□2 2찻술 2 teaspoon<br>□3 3찻술 3 teaspoon
Added cream to tea or coffee 차에 넣는 프림	□1	□2	□3	□4	□5	□6	□7	□8	□9	□1 1찻술 1 teaspoon<br>□2 2찻술 2 teaspoon<br>□3 3찻술 3 teaspoon
Green tea 녹차	□1	□2	□3	□4	□5	□6	□7	□8	□9	□1 ½잔 1/2 cup<br>□2 1잔 1 cup<br>□3 2잔 2 cups
청량음료 Soft drink (콜라, 사이다)	□1	□2	□3	□4	□5	□6	□7	□8	□9	□1 ½컵 (100ml) 1/2 cup (100ml)<br>□2 1컵 (200ml) 1 cup (200ml)<br>□3 2컵 (400ml) 2 cups (400ml)
기타음료 Other beverages (유자차, 매실차, 알로에, 수정과, 인삼차, 식혜, 대추차, 쌍화차 등)	□1	□2	□3	□4	□5	□6	□7	□8	□9	□1 ½잔 1/2 cup<br>□2 1잔 1 cup<br>□3 2잔 2 cups

For the following foods, please fill in number of months you consumed the foods, and the amount consumed per occassion during these months. If you consume only one season, then please mark 3 (3 months); two seasons, then mark 6 (6 months), three seasons, then mark 9 (9 months); four seasons, then plese mark 12 (12 months)

다음에 제시하는 식품은 **섭취하는 달수를 꼭✕ 표시**하시고, 섭취하는 계절동안의 섭취빈도를 표시해 주세요.
한 계절만 섭취하시면 ③, 두 계절 동안 섭취하시면 ⑥, 세 계절 동안 섭취하시면 ⑨, 사계절동안 섭취하시면 ⑫ 에 표시를 해 주십시오.

Usual consumption frequency during the season

	섭취 개월 (Number of months consumed)	섭취하는 계절동안의 섭취빈도									평균 1회 섭취분량 (Amount consumed per occasion)
		거의 안먹음 (Rarely)	월 (Monthly) 1회 (once)	월 (Monthly) 2~3회 (2-3 times)	주 (Weekly) 1~2회 (1-2 times)	주 (Weekly) 3~4회 (3-4 times)	주 (Weekly) 5~6회 (5-6 times)	일 (Daily) 1회 (once)	일 (Daily) 2회 (twi ce)	일 (Daily) 3회 (3 times)	
Strawberry 딸기	③ 3개월 (3 months) ⑥ 6개월 (6 months) ⑨ 9개월 (9 months) ⑫ 12개월 (12 months)	□1	□2	□3	□4	□5	□6	□7	□8	□9	□1 중 5개 medium size 5 items<br>□2 중 10개 medium size 10 items<br>□3 중 15개 medium size 15 items
Oriental melon / Melon 참외/ 메론	③ 3개월 ⑥ 6개월 ⑨ 9개월 ⑫ 12개월	□1	□2	□3	□4	□5	□6	□7	□8	□9	□1 중 1쪽 medium size 1 slice<br>□2 중 2쪽 medium size 2 slices<br>□3 중 4쪽 medium size 4 slices
Watermelon 수박	③ 3개월 ⑥ 6개월 ⑨ 9개월 ⑫ 12개월	□1	□2	□3	□4	□5	□6	□7	□8	□9	□1 중 ½쪽 medium size 1 slice<br>□2 중 1쪽 medium size 1/2 slice<br>□3 중 2쪽 medium size 2 slices
Peach / Plum 복숭아/ 자두	③ 3개월 ⑥ 6개월 ⑨ 9개월 ⑫ 12개월	□1	□2	□3	□4	□5	□6	□7	□8	□9	□1 중 ¼개/중 ½개 medium size 1/4 item/ 1/2 item<br>□2 중 ½개/중 1개 medium size 1/2 item/ 1 item<br>□3 중 1개/중 2개 medium size 1 item/ 2 items
Banana 바나나	③ 3개월 ⑥ 6개월 ⑨ 9개월 ⑫ 12개월	□1	□2	□3	□4	□5	□6	□7	□8	□9	□1 중 ½개 medium size 1/2 item<br>□2 중 1개 medium size 1 item<br>□3 중 2개 medium size 2 items
Persimmon / Dried persimmom 감/ 곶감	③ 3개월 ⑥ 6개월 ⑨ 9개월 ⑫ 12개월	□1	□2	□3	□4	□5	□6	□7	□8	□9	□1 중 ½개 medium size 1/2 item<br>□2 중 1개 medium size 1 item<br>□3 중 2개 medium size 2 items
Tangerine 귤	③ 3개월 ⑥ 6개월 ⑨ 9개월 ⑫ 12개월	□1	□2	□3	□4	□5	□6	□7	□8	□9	□1 중 ½개 medium size 1/2 item<br>□2 중 1개 medium size 1 item<br>□3 중 2개 medium size 2 items
Pear / Pear juice 배/ 배즙	③ 3개월 ⑥ 6개월 ⑨ 9개월 ⑫ 12개월	□1	□2	□3	□4	□5	□6	□7	□8	□9	□1 중 ¼개 medium size 1/4 item<br>□2 중 ⅓개 medium size 1/3 item<br>□3 중 ½개 medium size 1/2 item
Apple / Apple juice 사과/ 사과주스	③ 3개월 ⑥ 6개월 ⑨ 9개월 ⑫ 12개월	□1	□2	□3	□4	□5	□6	□7	□8	□9	□1 ¼개/ ½컵 (100ml) 1/4 item/ 1/2 cup (100ml)<br>□2 ½개/ 1컵 (200ml) 1/2 item/ 1 cup (200ml)<br>□3 1개/ 2컵 (400ml) 1 item/ 2 cups (400ml)
Orange / Orange juice 오렌지/ 오렌지주스	③ 3개월 ⑥ 6개월 ⑨ 9개월 ⑫ 12개월	□1	□2	□3	□4	□5	□6	□7	□8	□9	□1 중 ½개/½컵 (100ml) medium size 1/2 item/ 1/2 cup (100ml)<br>□2 중 1개/1컵 (200ml) medium size 1 item/ 1 cup (200ml)<br>□3 중 2개/2컵 (400ml) medium size 2 items/ 2 cups (400ml)
Grape / Grape juice 포도/ 포도주스	③ 3개월 ⑥ 6개월 ⑨ 9개월 ⑫ 12개월	□1	□2	□3	□4	□5	□6	□7	□8	□9	□1 ¼송이/½컵 (100ml) 1/4 item/ 1/2 cup (100ml)<br>□2 ½송이/1컵 (200ml) 1/2 item/ 1cup (200ml)<br>□3 1송이/2컵 (400ml) 1 item/ 2 cups (400ml)
Tomato / Cherry tomato / Tomato juice 토마토/ 방울토마토 / 토마토주스	③ 3개월 ⑥ 6개월 ⑨ 9개월 ⑫ 12개월	□1	□2	□3	□4	□5	□6	□7	□8	□9	□1 ½개/10개/½컵 (100ml) 1/2 item/ 10 item/ 1/2 cup (100ml)<br>□2 1개/20개/1컵 (200ml) 1 item/ 20 items/ 1 cup (200ml)<br>□3 2개/30개/2컵 (400ml) 2 items/ 30 items/ 2 cups (400ml)

질병관리본부
